# Asymmetric Brønsted acid-catalyzed aza-Diels–Alder reaction of cyclic *C*-acylimines with cyclopentadiene

**DOI:** 10.3762/bjoc.8.208

**Published:** 2012-10-23

**Authors:** Magnus Rueping, Sadiya Raja

**Affiliations:** 1 Institute of Organic Chemistry, RWTH Aachen University, Landoltweg 1, D-52074 Aachen, Germany

**Keywords:** BINOL phosphate, [4 + 2] cycloaddition, Diels–Alder reaction, organocatalysis

## Abstract

A new chiral Brønsted acid-catalyzed aza-Diels–Alder reaction of cyclic *C*-acylimines with cyclopentadiene has been developed. The reaction provides optically active aza-tetracycles in good yields with high diastereo- and enantioselectivities under mild reaction conditions.

## Introduction

**Scheme 1:**
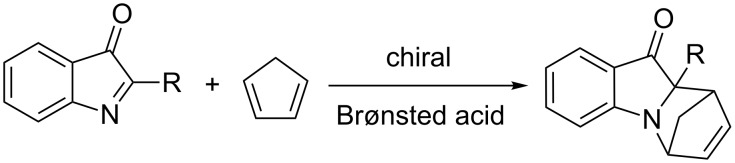
Brønsted acid catalyzed aza-Diels–Alder reaction of cyclic *C*-acylimines with cyclopentadiene.

The enantioselective aza-Diels–Alder reaction is an important method for the construction of optically active, nitrogen-containing, six-membered rings, such as tetrahydroquinolines and piperidines. N-heterocycles are found in a wide range of natural products and many biologically active compounds [1–4]. To date, most aza-asymmetric Diels–Alder reactions have been catalyzed by chiral Lewis acids [5–16]. Recently, chiral Brønsted acids have attracted interest as effective catalysts for a variety of asymmetric transformations involving imine electrophiles [17–23]. Among others, the aza-Diels–Alder reaction of imino-dienophiles has been investigated and it was shown that the reaction between arylimines and dienes, catalyzed by chiral Brønsted acids, proceeds with high levels of enantioselectivity. However, these reactions are limited to electron-rich dienes including Brassard’s and Danishefsky dienes [24–32]. To the best of our knowledge the enantioselective Brønsted acid catalyzed aza-Diels–Alder reaction of imines with less-electron-rich dienes has not been reported. Thus, we decided to examine the unprecedented Brønsted acid catalyzed aza-Diels–Alder reaction of cyclic *C*-acylimines with cyclopentadiene providing optically active nitrogen-containing heterocycles (Scheme 1).

## Results and Discussion

Our initial study began with the examination of the the aza-Diels–Alder reaction of cyclic *C*-acylimine **1** with cyclopentadiene (**2**) in the presence of BINOL-derived phosphoric acid diesters and *N*-triflylphosphoramides **4–6** (Table 1) [33–51] as the catalysts. We were delighted to see that the reaction proceeded smoothly at different temperatures and that the product could be obtained with an enantiomeric excess of 8% ee when the reaction was performed in toluene at −60 °C in the presence of catalyst **4a** (Table 1, entry 1). A slight increase in enantioselectivity was observed when the reaction was conducted at −78 °C (Table 1, entry 2). Subsequently, different catalysts were applied in the Brønsted acid catalyzed hetero-Diels–Alder reaction. From the different catalysts tested, phosphoric acid diester **4b**, with the 2,4,6-triisopropylphenyl substituent in the 3,3’-position of the BINOL backbone, proved to be the best catalyst, and the product was obtained with an encouraging enantiomeric excess of 74% (Table 1, entry 3). To optimize the reaction conditions further we evaluated the catalyst loading and solvent. However, the reduction of catalyst loading from 5 to 1 mol % resulted in a significant decrease in enantioselectivity (Table 1, entries 8 and 9).

**Table 1:** Optimization of reaction conditions^a^.

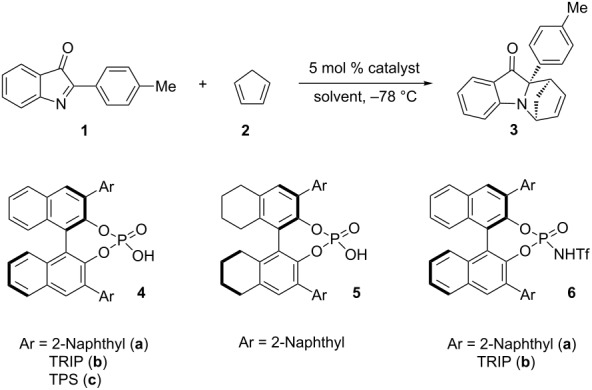					

entry	catalyst	x mol %	solvent	*t* [min]	ee [%]^b, c^

1^d^	**4a**	5	toluene	2	8
2	**4a**	5	toluene	15	16
3	**4b**	5	toluene	20	74
4	**4c**	5	toluene	8h	40
5	**5**	5	toluene	90	16
6	**6a**	5	toluene	40	20
7	**6b**	5	toluene	20	60
8	**4b**	2	toluene	60	60
9	**4b**	1	toluene	60	43
10	**4b**	5	toluene:CHCl_3_ 1:1	40	13
11	**4b**	5	toluene:CH_2_Cl_2_ 1:1	10	20
12	**4b**	5	toluene:hexane 1:1	5 h	74
13	**4b**	5	toluene:hexane 1:2	6 h	90
14	**4b**	5	toluene:hexane 1:3	8 h	94
15	**4b**	5	toluene:hexane 1:4	16 h	94

^a^Reaction conditions: Imine **1**, cyclopentadiene (2.0 equiv) and catalyst. ^b^Enantiomeric excess was determined by HPLC on a chiral phase. ^c^Only one diastereomer is formed. ^d^The reaction was carried out at −60 °C.

In our previous studies in asymmetric Brønsted acid catalysis, we noticed that solvent mixtures can strongly influence both the reactivity and selectivity. Thus, we evaluated different solvent mixtures. When a 1:1 mixture of toluene and CHCl_3_ was used the enantioselectivity dropped considerably. The same effect was observed when a mixture of toluene and CH_2_Cl_2_ was used (Table 1, entries 10 and 11). Hence, the chlorinated solvents were replaced by hexane. Interestingly, use of a 1:1 mixture of toluene and hexane afforded the corresponding product without loss of selectivity, but, as anticipated, the reaction time was longer (Table 1, entry 12). Pleasingly, when the reaction was carried out in a 2:1 mixture of hexane/toluene the product exhibited excellent enantioselectivity (Table 1, entry 13). Further improvement of selectivity was obtained by increasing the hexane/toluene ratio to 3:1, which delivered the product with an excellent enantiomeric excess of 94% (Table 1, entry 14). With the optimal reaction conditions in hand, the substrate scope of the aza-Diels–Alder reaction was examined (Table 2). Various substituted cyclic *C*-acylimines **1a–i** with electron-donating and electron-withdrawing groups, as well as different substitutions patterns, were applied. In all cases the corresponding tetracyclic products were obtained in high yields and with excellent diastereo- and enantioselectivities. However, the use of less reactive dienes including cyclohexadiene or linear 1,3-pentadienes resulted in reduced product formation or provided the desired products with low enantioselectivities [52–55].

**Table 2:** Scope of the aza-Diels–Alder reaction^a^.

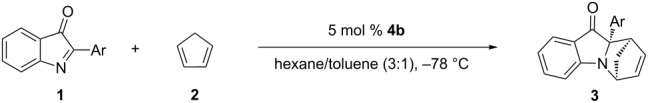				

entry	product	*t* [h]	yield [%]^b^	ee [%]^c, d^

1	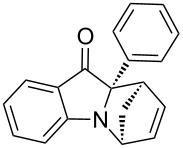 **3a**	3	92	89
2	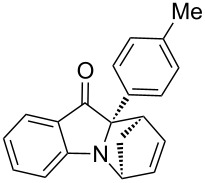 **3b**	8	86	94
3	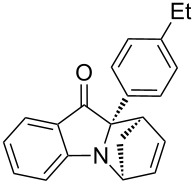 **3c**	2	83	86
4	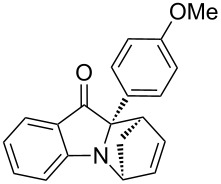 **3d**	4	79	90
5	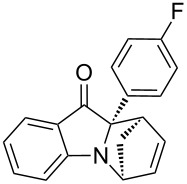 **3e**	8	73	91
6	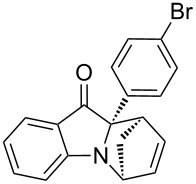 **3f**	3	94	82
7	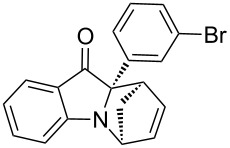 **3g**	48	83	84
8	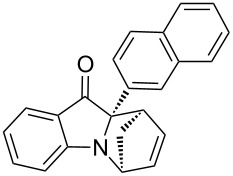 **3h**	96	79	91
9	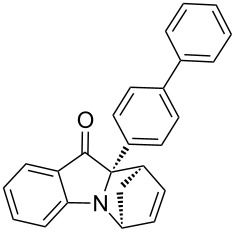 **3i**	96	83	86

^a^Reaction conditions: Imine **1**, cyclopentadiene (2.0 equiv) and 5 mol % **4b**. ^b^Yield of the isolated product after column chromatography. ^c^The enantiomeric excess was determined by HPLC on a chiral phase. ^d^Only one diastereomer is formed.

## Conclusion

In conclusion, we have developed an enantioselective Brønsted acid catalyzed aza-Diels–Alder reaction of *C*-acylimines with cyclopentadiene. The corresponding aza-tetracycles were obtained in high yields and with excellent enantio- and diastereoselectivities under mild reaction conditions. The results reported not only show that chiral BINOL derived phosphoric acid diesters can be efficient catalysts for [4 + 2] cycloadditions involving less-electron-rich dienes but additionally demonstrate the high potential of these acidic Brønsted acids in asymmetric catalysis.

## Experimental

The starting materials **1a–i** were synthesized according to a known literature procedure [56].

General procedure for the aza-Diels–Alder reaction: In a typical experiment the imine and cyclopentadiene were suspended in a mixture of hexane/toluene (3:1) in a screw-capped test tube and stirred at −78 °C for 10 min. The catalyst (5 mol %) was added to the solution and the mixture was stirred until consumption of the imine. The crude reaction mixture was directly charged on silica gel and purified by column chromatography (hexane/ethyl acetate as eluent) to afford the desired products.

## Supporting Information

File 1 Experimental details and characterization of the synthesized compounds.
