# Supplementary material for: Asymmetric Brønsted acid-catalyzed aza-Diels–Alder reaction of cyclic C-acylimines with cyclopentadiene
Source: Beilstein J Org Chem. 2012 Oct 23;8:1819–24. doi: 10.3762/bjoc.8.208 (PMC3511017; doi:10.3762/bjoc.8.208)
Supplement: File 1 — Experimental details and characterization of the synthesized compounds. [file Beilstein_J_Org_Chem-08-1819-s001.pdf]

Supporting Information

for

Asymmetric Brønsted acid-catalyzed aza-Diels–Alder reaction of cyclic *C*-acylimines with cyclopentadiene

Magnus Rueping* and Sadiya Raja

Address: Institute of Organic Chemistry, RWTH Aachen University, Landoltweg 1, D-52074 Aachen, Germany

Email: Magnus Rueping* - magnus.rueping@rwth-aachen.de

* Corresponding author

Experimental details and characterization of the synthesized compounds

General: All commercially available compounds were purified before use by distillation or crystallization. Solvents for chromatography were technical grade and distilled prior to use. Analytical thin-layer chromatography (TLC) was performed on Merck silica gel aluminium plates with F-254 indicator, visualised by irradiation with UV light. Column chromatography was performed by using silica gel Merck 60 (particle size 0.040-0.063

mm). ^{1}H NMR and ^{13}C NMR were recorded on a Mercury 300 or Inova 400 spectrometer in $CDCl_3$. Data are reported in the following order: chemical shift (δ) in ppm; multiplicities are indicated s (singlet), bs (broad singlet), d (doublet), t (triplet), m (multiplet); coupling constants (*J*) are in hertz (Hz). Mass spectra (MS-EI, 70 eV) were conducted on GC-MS Shimadzu QP2010 (column: Equity®-5, length × I.D. 30 m × 0.25 mm, df 0.25 μm, lot # 28089-U, Supelco) IR spectra were recorded on a Jasco FT/IR-420 spectrometer and are reported in terms of frequency of absorption (cm^{-1}). Optical rotations were measured on a Perkin Elmer 241 polarimeter. The enantiomeric excesses were determined by HPLC analysis using a chiral stationary-phase column (column, Daicel Co. CHIRALCEL AD-H; eluent: *n*-hexane/2-propanol). The chiral HPLC methods were calibrated with the corresponding racemic mixtures. Chemical yields refer to pure isolated substances.

General procedure for the aza-Diels–Alder reaction: In a typical experiment the imine and cyclopentadiene were suspended in a mixture of hexane/toluene (3:1) in a screw-capped test tube and stirred at −78 °C for 10 min. The catalyst (5 mol %) was added to the solution and the mixture was stirred until consumption of the imine. The crude reaction mixture was directly charged on silica gel and purified by column chromatography (hexane/ethylacetate as eluent) to afford the desired products.

2-Phenylindol-3-one (1a)

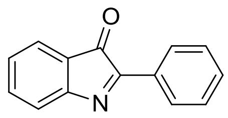

The product was purified by column chromatography (eluent EtOAc/hexane 1:40). Red solid, mp: 102–104 °C; ^{1}H NMR ($CDCl_3$, 300 MHz) δ 8.40–8.37 (m, 2H), 7.57–

7.41 (m, 6H), 7.28–7.23 (m, 1H) ppm; ^{13}C NMR ($CDCl_3$, 75 MHz) δ 193.3, 160.8, 159.5, 136.8, 132.2, 129.8, 129.3, 128.8, 128.3, 124.7, 123.0, 121.9 ppm; IR (KBr) $\tilde{\nu}$: 3430, 3067, 3028, 2324, 2111, 1751, 1721, 1597, 1536, 1447, 1253, 1163, 1076, 871, 763, 865 cm^{-1}; EIMS *m/z* (%): 207.1 (41) [M]$^{+\bullet}$, 179.1 (100), 76.2 (47).

2-*p*-Tolylindol-3-one (1b)

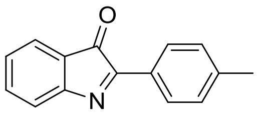

The product was purified by column chromatography (eluent EtOAc/hexane 1:40). Red solid, mp: 125–128 °C; ^{1}H NMR ($CDCl_3$, 300 MHz) δ 8.30–8.28 (m, 2H), 7.55–7.51 (m, 2H), 7.41–7.39 (m, 1H), 7.31–7.29 (m, 2H), 7.24–7.22 (m, 1H), 2.42 (s, 3H) ppm; ^{13}C NMR ($CDCl_3$, 75 MHz) δ 193.5, 160.7, 160.7, 159.7, 142.9, 136.6, 129.5, 129.2, 127.9, 124.5, 123.0, 121.6, 21.8 ppm; IR (KBr) $\tilde{\nu}$: 3423, 3032, 2921, 2323, 2080, 1996, 1925, 1758, 1720, 1601, 1537, 1506, 1450, 1315, 1255, 1165, 1042, 872, 831, 793, 761, 706, 658 cm^{-1}; EIMS *m/z* (%): 221.1 (56) [M]$^{+\bullet}$, 193.1 (100), 165.1 (26), 116.1 (23).

2-(4-Ethylphenyl)indol-3-one (1c)

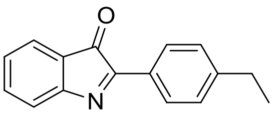

The product was purified by column chromatography (eluent EtOAc/hexane 1:40). Red solid, mp: 127–130 °C; ^{1}H NMR ($CDCl_3$, 300 MHz) δ 8.31–8.29 (m, 2H), 7.53–7.51 (m, 2H), 7.39–7.37 (m, 1H), 7.32–7.30 (m, 2H), 7.24–7.20 (m, 1H), 2.74–2.68 (m,

2H), 1.87 (m, 1H) 1.29–1.25 (t, J = 7.6 Hz, 3H) ppm; ^{13}C NMR ($CDCl_3$, 75 MHz) δ 193.6, 160.7, 159.7, 149.0, 136.6, 129.2, 128.3, 127.9, 127.3, 124.5, 123.0, 121.6, 29.1, 15.2 ppm; IR (KBr) $\tilde{\nu}$: 3418, 3052, 2962, 2323, 2093, 1923, 1755, 1723, 1602, 1571, 1537, 1506, 1452, 1315, 1256, 1167, 1054, 1015, 960, 874, 839, 800, 763, 714, 657 cm^{-1}; EIMS m/z (%): 235.1 (84) $[M]^{+\bullet}$, 207.2 (100), 192 (30).

2-(4-Methoxyphenyl)indol-3-one (1d)

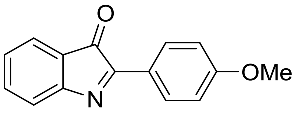

The product was purified by column chromatography (eluent EtOAc/hexane 1:40). Red solid, mp:114–117 °C; ^{1}H NMR ($CDCl_3$, 300 MHz) δ 8.37–8.35 (m, 2H), 7.50–7.45 (m, 2H), 7.33–7.30 (m, 1H), 7.20–7.15(m, 1H), 6.97–6.94 (m, 2H), 3.8 (s, 3H) ppm; ^{13}C NMR ($CDCl_3$, 75 MHz) δ 194.1, 162.9, 160.2, 160.1, 136.6, 131.2, 127.5, 124.4, 123.1, 122.6, 121.4, 114.3, 55.3 ppm; IR (KBr) $\tilde{\nu}$: 3421, 3002, 2918, 2568, 2097, 1757, 1717, 1596, 1534, 1498, 1452, 1421, 1308, 1253, 1160, 1048, 1020, 948, 871, 842, 795, 755, 711, 658 cm^{-1}; EIMS m/z (%): 237.2(1) $[M]^{+\bullet}$, 133.1 (25), 104.1 (26), 76.2 (100).

2-(4-Fluorophenyl)indol-3-one (1e)

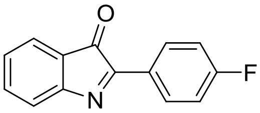

The product was purified by column chromatography (eluent EtOAc/hexane 1:40). Red solid, mp: 127–129 °C; ^{1}H NMR ($CDCl_3$, 400 MHz) δ 8.42–8.38 (m, 2H), 7.54–

7.50 (m, 2H), 7.39–7.37 (m, 1H), 7.25–7.22 (m, 1H), 7.18–7.14 (m, 2H) ppm; ^{13}C NMR ($CDCl_3$, 75 MHz) δ 193.1, 166.4, 163.9, 159.7, 159.4, 136.7, 131.5, 131.4, 128.2, 126.1, 124.6, 122.9, 121.8, 116.1, 115.9 ppm; IR (KBr) $\tilde{\nu}$: 3412, 3062, 2924, 2323, 2111, 1920, 1753, 1714, 1595, 1540, 1501, 1451, 1415, 1358, 1299, 1258, 1227, 1157, 1101, 1078, 1044, 1013, 947, 872, 843, 799, 757, 707, 659 cm^{-1}; EIMS *m/z* (%): 225.0 (46) $[M]^{+\bullet}$, 197.0 (100), 170.1 (12), 76.2 (38).

2-(4-Bromophenyl)indol-3-one (1f)

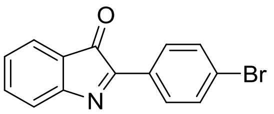

The product was purified by column chromatography (eluent EtOAc/hexane 1:60). Red solid, mp: 138–140 °C; ^{1}H NMR ($CDCl_3$, 300 MHz) δ 8.24–8.22 (m, 2H), 7.60–7.58 (m, 2H), 7.53–7.49 (m, 2H), 7.38–7.37 (m, 1H), 7.24–7.22 (m, 1H) ppm; ^{13}C NMR ($CDCl_3$, 75 MHz) δ 192.9, 160.1, 159.4, 136.8, 132.0, 130.5, 128.8, 128.5, 127.3, 124.7, 122.9, 122.0 ppm; IR (KBr) $\tilde{\nu}$: 3435, 3070, 2851, 2323, 2110, 1937, 1749, 1723, 1603, 1580, 1535, 1478, 1453, 1397, 1283, 1258, 1162, 1067, 1004, 871, 844, 795, 700, 655 cm^{-1}; EIMS *m/z* (%): 285.0 (60) $[M]^{+\bullet}$, 259.0 (77), 178.1 (100), 151.1, (50), 102.1 (41), 76.2 (88).

2-(3-Bromophenyl)indol-3-one (1g)

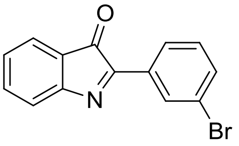

The product was purified by column chromatography (eluent EtOAc/hexane 1:5). Red solid, mp: 124–127 °C; ^{1}H NMR ($CDCl_3$, 400 MHz) δ 7.34–7.12 (m, 5H), 5.68 (bs, 1H), 3.80 (d, *J* =6.5, 1H) ppm; ^{13}C NMR ($CDCl_3$, 62.5 MHz) δ 202.2, 149.5, 145.9, 141.7, 128.9, 127.3, 127.1, 44.9, 42.3, 12.6 ppm; IR (KBr) $\tilde{\nu}$: 3332, 2918, 2853, 1693, 1511, 1403, 1351, 1122, 928, 814 cm^{-1}; EIMS *m/z* (%): 188.2 (100) $[M]^{+\bullet}$.

2-(Naphthalen-2-yl)indol-3-one (1h)

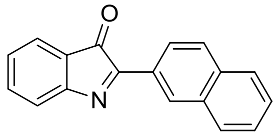

The product was purified by column chromatography (eluent EtOAc/hexane 1:60). Red solid, mp: 126–130; ^{1}H NMR ($CDCl_3$, 300 MHz) δ 9.01 (s, 1H), 8.38–8.35 (m, 1H), 7.99–7.96 (m, 1H), 7.92–7.90 (m, 1H), 7.85–7.84 (m, 1H), 7.59–7.51 (m, 4H), 7.44–7.42 (m, 1H), 7.27–7.24 (m, 1H) ppm; ^{13}C NMR ($CDCl_3$, 75 MHz) δ 193.4, 160.6, 159.6, 136.7, 136.5, 134.9, 132.9, 131.3, 129.6, 128.6, 128.2, 128.2, 127.7, 126.6, 124.6, 121.9 ppm; IR (KBr) $\tilde{\nu}$: 3421, 3053, 2296, 2114, 1758, 1716, 1529, 1453, 1349, 1269, 1163, 1128, 1010, 948, 904, 863, 821, 799, 746, 713, 656 cm^{-1}; EIMS *m/z* (%): 257.1 (81) $[M]^{+\bullet}$, 229.1 (100), 153.1 (43), 127.1 (21).

2-(Biphenyl-4-yl)indol-3-one (1i)

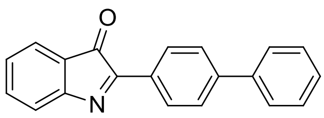

The product was purified by column chromatography (eluent EtOAc/hexane 1:60). Red solid, mp: 100–103 °C; ^{1}H NMR ($CDCl_3$, 300 MHz) δ 8.40–8.38 (m, 2H), 7.65–

7.63 (m, 2H), 7.58–7.57 (m, 2H), 7.48–7.44 (m, 2H), 7.41–7.29 (m, 4H), 7.19–7.15 (m, 1H) ppm; ^{13}C NMR (CDCl$_3$, 75 MHz) δ 193.4, 160.4, 159.6, 144.6, 139.8, 136.7, 129.7, 128.8, 128.6, 128.1, 128.0, 127.3, 127.0, 124.6, 123.0, 121.8 ppm; IR (KBr) $\tilde{\nu}$: 3341, 3073, 2089, 1723, 1677, 1599, 1484, 1402, 1358, 1261, 1176, 1119, 1003, 957, 875, 836, 762, 720, 687 cm^{-1}; EIMS *m/z* (%): 283.2 (10) [M]$^{+\bullet}$, 255.2 (14), 196.2 (85), 181.2 (100), 152.2 (66).

10-Phenyl-2-azatetracyclo[9.2.1.0^{2,10}.0^{3,8}]tetradeca-3(8),4,6,12-tetraen-9-one (3a)

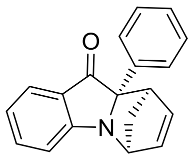

The product was purified by column chromatography (eluent EtOAc/hexane 1:10). Yield: 92%; yellow oil; ^{1}H NMR (CDCl$_3$, 400 MHz) δ 7.99–7.96 (m, 2H), 7.59–7.50 (m, 4H), 7.45–7.42 (m, 1H), 7.24–6.21 (m, 1H), 7.02–6.96 (m, 1H), 6.30–6.26 (m, 1H), 5.86–5.83 (m, 1H), 4.73 (bs, 1H), 3.61 (bs, 1H), 2.01–1.97 (m, 1H), 1.80–1.76 (m, 1H) ppm; ^{13}C NMR (CDCl$_3$, 75 MHz) δ 204.4, 164.8, 139.6, 136.5, 134.6, 134.2, 129.3, 128.3 (2x), 127.5, 126.8 (2x), 124.7, 122.5, 117. 3, 78.6, 65.5, 53.1, 49.7 ppm; IR (KBr) $\tilde{\nu}$: 3434, 3016, 1919, 1701, 1607, 1468, 1303, 1215, 756, 697 cm^{-1}; EIMS *m/z* (%): 273.1 (78) [M]$^{+}$, 244.1 (33), 208.1 (40), 179.1 (100); $[\alpha]_{D}^{RT}$ = −245 (*c* 6.5, MeOH); HPLC conditions: AD-H column, *n*-hexane/2-propanol = 90/10, flow rate = 0.6 mL·min^{-1}, major enantiomer: t_R = 12.36 min; minor enantiomer: t_R = 21.32 min.

10-(4-Methylphenyl)-2-azatetracyclo[9.2.1.0^{2,10}.0^{3,8}]tetradeca-3(8),4,6,12-tetraen-9-one (3b)

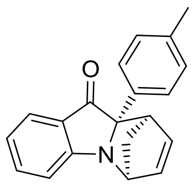

The product was purified by column chromatography (eluent EtOAc/hexane 1:10). Yield: 86%; yellow oil; ^{1}H NMR ($CDCl_3$, 400 MHz) δ 7.73–7.68 (m, 2H), 7.53–7.47 (m, 1H), 7.44–7.40 (m, 1H), 7.21–7.18 (m, 1H), 7.15–7.12 (m, 2H), 6.98–6.93 (m, 1H), 6.28–6.25 (m, 1H), 5.83–5.80 (m, 1H), 4.70 (bs, 1H), 3.60 br (s, 1H), 2.31 (s, 3H), 2.07–2.04 (m, 1H), 1.78–1.74 (m, 1H) ppm; ^{13}C NMR ($CDCl_3$, 75 MHz) δ 204.5, 164.8, 137.2, 136.6, 136.4, 134.5, 134.2, 129.3, 129.1 (2x), 126.7 (2x), 124.7, 122.4, 117.2, 78.4, 65.5, 52.9, 49.7, 21.0 ppm; IR (KBr) $\tilde{\nu}$: 3376, 3005, 2871, 1762, 1702, 1607, 1465, 1319, 1107, 695, 796, 670 cm^{-1}; EIMS *m/z* (%): 287.1 (93) [M]$^{+}$, 258.1 (24), 221.1 (51), 193.1 (100); $[\alpha]_{D}^{RT}$ = −209 (*c* 8.8, MeOH); HPLC conditions: AD-H column, *n*-hexane/2-propanol = 90/10, flow rate = 0.6 mL·min^{-1}, major enantiomer: t_R = 12.10 min; minor enantiomer: t_R = 28.57 min.

10-(4-Ethylphenyl)-2-azatetracyclo[9.2.1.0^{2,10}.0^{3,8}]tetradeca-3(8),4,6,12-tetraen-9-one (3c)

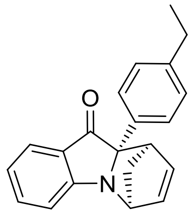

The product was purified by column chromatography (eluent EtOAc/hexane 1:10). Yield: 83%; yellow oil; ^{1}H NMR ($CDCl_3$, 400 MHz) δ 7.75–7.71 (m, 2H), 7.53–7.47 (m, 1H), 7.43–7.40 (m, 1H), 7.22–7.14 (m, 3H), 6.98–6.93 (m, 1H), 6.28–6.25 (m, 1H),

5.83–5.80 (dd, J = 5.5, 2.0 Hz, 1H), 4.71–4.69 (m, 1H), 3.62–3.59 (m, 1H), 2.65–2.57 (m, 2H), 2.07 (d, J = 8.8 Hz, 1H), 1.75 (d, J = 8.7 Hz, 1H), 1.21 (t, J = 15.2, 7.6 Hz, 1H) ppm; ^{13}C NMR ($CDCl_3$, 75 MHz) δ 204.6, 164.8, 143.5, 136.8, 136.4, 134.5, 134.2, 129.4, 127.9, 126.7, 124.7, 122.4, 117. 3, 78.5, 65.5, 52.9, 49.7, 28.4, 15.5 ppm; IR (KBr) $\tilde{\nu}$: 3392, 2963, 1704, 1606, 1465, 1303, 1107, 967, 758, 691 cm^{-1}; EIMS *m/z* (%): 301.1 (64) [M]$^+$, 272.1 (54), 235.1 (25), 207.1 (100); $[\alpha]_D^{RT}$ = −208 (*c* 16.4, MeOH); HPLC conditions: AD-H column, *n*-hexane/2-propanol = 90/10, flow rate = 0.6 mL·min^{-1}, major enantiomer: t_R = 12.15 min; minor enantiomer: t_R = 24.65 min.

10-(4-Methoxyphenyl)-2-azatetracyclo[9.2.1.0^{2,10}.0^{3,8}]tetradeca-3(8),4,6,12-tetraen-9-one (3d)

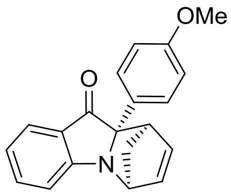

The product was purified by column chromatography (eluent EtOAc/hexane 1:10). Yield: 79%; red oil; ^{1}H NMR ($CDCl_3$, 400 MHz) δ 7.53–7.41 (m, 4H), 7.27–7.18 (m, 2H), 6.98–6.93 (t, J = 7.2 Hz, 1H), 6.82–6.78 (m, 1H), 6.28–6.25 (m, 1H), 5.84–5.81 (m, 1H), 4.69 (bs, 1H), 3.81 (s, 3H), 3.61 (bs, 1H), 2.07 (d, J = 8.8 Hz, 1H), 1.76 (d, J = 8.8 Hz, 1H) ppm; ^{13}C NMR ($CDCl_3$, 75 MHz) δ 204.3, 164.8, 159.8, 141.2, 136.5, 134.7, 134.2, 129.3 (2x), 124.7 (2x), 122.5, 119.2, 117.3, 117.3, 112.8, 112.7, 78.6, 65.5, 55.2, 53.1, 49.7 ppm; IR (KBr) $\tilde{\nu}$: 3394, 3009, 2920, 2850, 1702, 1605, 1507, 1464, 1302, 1251, 1173, 1033, 756, 528 cm^{-1}; EIMS *m/z* (%): 303.0 (58) [M]$^+$, 237.0 (82), 209.0 (100); $[\alpha]_D^{RT}$ = −151 (*c* 6.3, MeOH); HPLC conditions: AD-H column, *n*-hexane/2-propanol = 90/10, flow rate = 0.6 mL·min^{-1}, major enantiomer: t_R = 17.02 min; minor enantiomer: t_R = 41.32 min.

10-(4-Fluorophenyl)-2-azatetracyclo[9.2.1.0^{2,10}.0^{3,8}]tetradeca-3(8),4,6,12-tetraen-9-one (3e)

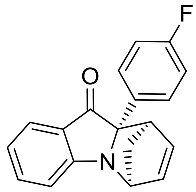

The product was purified by column chromatography (eluent EtOAc/hexane 1:10). Yield: 73%; yellow oil; ^{1}H NMR ($CDCl_3$, 400 MHz) δ 7.82–7.79 (m, 2H), 7.53–7.50 (m, 1H), 7.44–7.42 (m, 1H), 7.21–7.19 (m, 1H), 7.02–6.96 (m, 3H), 6.27–6.25 (m, 1H), 5.82–5.81 (dd, *J* = 5.4, 1.9 Hz, 1H), 4.70 (d, *J* = 1.2 Hz, 1H), 3.56 (bs, 1H), 2.02 (d, *J* = 8.7 Hz, 1H), 1.77 (d, *J* = 8.7 Hz, 1H) ppm; ^{13}C NMR ($CDCl_3$, 75 MHz) δ 204.2, 164.7, 163.2, 161.6, 136.7, 134.7, 134.1, 129.7, 128.5, 128.5, 124.8, 122.6, 117.3, 115.2, 115.1, 78.0, 65.6, 53.2, 49.7 ppm; IR (KBr) $\tilde{\nu}$: 3394, 3068, 2997, 2860, 1704, 1605, 1503, 1316, 1223, 1156, 1105, 965, 912, 764, 733, 519 cm^{-1}; EIMS *m/z* (%): 291.1 (56) $[M]^+$, 262.1 (35), 225.1 (83), 197.1 (100) ; $[\alpha]_D^{RT}$ = −229 (*c* 6.7, MeOH); HPLC conditions: AD-H column, *n*-hexane/2-propanol = 90/10, flow rate = 0.6 mL·min^{-1}, major enantiomer: t_R = 11.64 min; minor enantiomer: t_R = 21.06 min.

10-(4-Bromophenyl)-2-azatetracyclo[9.2.1.0^{2,10}.0^{3,8}]tetradeca-3(8),4,6,12-tetraen-9-one (3f)

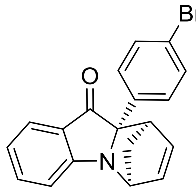

The product was purified by column chromatography (eluent EtOAc/hexane 1:10). Yield: 94%; yellow oil; ^{1}H NMR ($CDCl_3$, 400 MHz) δ 7.74–7.70 (m, 2H), 7.54–7.40 (m,

1H), 7.46–7.42 (m, 3H), 7.20 (d, *J* = 7.5 Hz, 1H), 6.99–6.96 (m, 1H), 6.27–6.24 (m, 1H), 5.83–5.81 (dd, *J* = 5.5, 2.0 Hz, 1H) 4.70 (bs, 1H), 3.55 (bs, 1H), 2.00 (d, *J* = 8.8 Hz, 1H), 1.77 (d, *J* = 8.8 Hz, 1H) ppm; ^{13}C NMR ($CDCl_3$, 75 MHz) δ 203.8, 164.7, 138.8, 136.728, 134.7, 134.1, 131.4 (2x), 129.1, 128.7 (2x), 124.8, 122.7, 121.7, 117.3, 78.6, 65.6, 53.2, 49.7 ppm; IR (KBr) $\tilde{\nu}$: 3395, 3004, 1701, 1600, 1473, 1305, 1260, 1194, 1106, 1063, 1007, 912, 796, 692 cm^{-1}; EIMS *m/z* (%): 352.7 (38) [M]$^+$, 284.7 (86), 258.7 (100), 177.9 (69), 150.8 (52); $[\alpha]_D^{RT}$ = −154 (*c* 5.8, MeOH); HPLC conditions: AD-H column, *n*-hexane/2-propanol = 90/10, flow rate = 0.6 mL·min^{-1}, major enantiomer: t_R = 12.99 min; minor enantiomer: t_R = 31.05 min.

10-(3-Bromophenyl)-2-azatetracyclo[9.2.1.0^{2,10}.0^{3,8}]tetradeca-3(8),4,6,12-tetraen-9-one (3g)

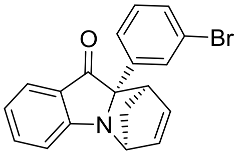

The product was purified by column chromatography (eluent EtOAc/hexane 1:10). Yield: 83%; orange oil; ^{1}H NMR ($CDCl_3$, 400 MHz) δ 8.00–7.98 (m, 1H), 7.79 (m, 1H), 7.54–7.51 (m, 1H), 7.44–7.42 (m, 1H), 7.39–7.37 (m, 1H), 7.22–7.19 (m, 2H), 6.99–6.97 (m, 1H), 6.26–6.25 (m, 1H), 5.83–5.82 (dd, *J* = 5.4, 1.8 Hz, 1H) 4.70 (bs, 1H), 3.56 (bs, 1H), 2.02 (d, *J* = 8.8 Hz, 1H), 1.77 (d, *J* = 8.8 Hz, 1H) ppm; ^{13}C NMR ($CDCl_3$, 75 MHz) δ 203.6, 164.7, 142.0, 136.8, 134.8, 134.0, 130.6, 130.0, 129.9, 129.1, 125.5, 124.8, 122.7, 122.4, 117.4, 78.1, 65.6, 53.3, 49.7 ppm; IR (KBr) $\tilde{\nu}$: 3391, 2996, 2854, 1705, 1605, 1467, 1302, 1197, 1104, 967, 912, 778, 695 cm^{-1}; EIMS *m/z* (%): 353.0 (73) [M]$^+$, 322.1 (27), 286.0 (98), 257.0 (100), 178.2 (93); $[\alpha]_D^{RT}$ = −268 (*c* 2.5, MeOH);

HPLC conditions: AD-H column, *n*-hexane/2-propanol = 90/10, flow rate = 0.6 mL·min^{-1}, major enantiomer: t_R = 11.53 min; minor enantiomer: t_R = 13.35 min.

10-(2-Naphthyl)-2-azatetracyclo[9.2.1.0^{2,10}.0^{3,8}]tetradeca-3(8),4,6,12-tetraen-9-one (3h)

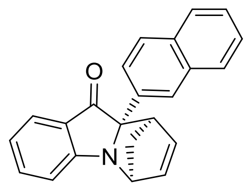

The product was purified by column chromatography (eluent EtOAc/hexane 1:10). Yield: 79%; orange oil; ^{1}H NMR ($CDCl_3$, 400 MHz) δ 8.30–8.29 (m, 1H), 7.97–7.95 (m, 1H), 7.83–7.77 (m, 3H), 7.53–7.49 (m, 1H), 7.53–7.49 (m, 1H), 7.44–7.39 (m, 3H) 7.26–7.24 (m, 1H), 6.98–6.94 (m, 1H), 6.32–6.92 (m, 1H), 5.85–5.83 (dd, *J* = 5.5, 2.0 Hz, 1H), 4.75–4.74 (m, 1H), 3.72 (bs, 1H), 2.08 (d, *J* = 8.8 Hz, 1H), 1.76 (d, *J* = 8.8 Hz, 1H) ppm; ^{13}C NMR (CDCl3, 75 MHz) δ 204.3, 164.8, 136.9, 136.5, 134.7, 134.2, 133.2, 132.9, 129.3, 128.1 (2x), 127.5, 125.9, 125.8, 125.8, 124.8, 124.8, 122.5, 117.3, 78.7, 65.6, 52.9, 49.7 ppm; IR (KBr) $\tilde{\nu}$: 3389, 3062, 2997, 2856, 1942, 1705, 1602, 1464, 1308, 1266, 1198, 1106, 1059, 967, 746, 684, 476 cm^{-1}; EIMS *m/z* (%): 322.9 (56) $[M]^+$, 256.9 (70), 229.0 (100), 152.9 (38); $[\alpha]_D^{RT}$ = −79 (*c* 4.8, MeOH); HPLC conditions: AD-H column, *n*-hexane/2-propanol = 90/10, flow rate = 0.6 mL·min^{-1}, major enantiomer: t_R = 28.69 min; minor enantiomer: t_R = 45.30 min.

10-[1,1'-Biphenyl-4-yl]-2-azatetracyclo[9.2.1.0^{2,10}.0^{3,8}]tetradeca-3(8),4,6,12-tetraen-9-one (3i)

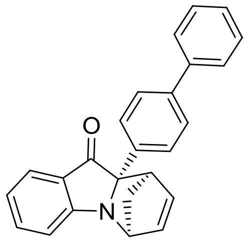

The product was purified by column chromatography (eluent EtOAc/hexane 1:10). Yield: 83%; yellowish oil; ^{1}H NMR ($CDCl_3$, 400 MHz) δ 7.92–7.89 (m, 1H), 7.58–7.40 (m, 8H), 7.35–7.30 (m, 1H), 7.00–6.96 (m, 1H), 6.31–6.28 (m, 1H), 5.86–5.84 (dd, *J* = 5.5, 2.0 Hz, 1H), 4.75–4.73 (m, 1H), 3.67–3.66 (m, 1H), 2.11 (d, *J* = 8.8 Hz, 1H), 1.80 (d, *J* = 8.8 Hz, 1H) ppm; ^{13}C NMR ($CDCl_3$, 75 MHz) δ 204.3, 164.8, 140.8, 140.32, 138.6, 136.5, 134.6, 134.2, 129.3, 128.9, 128.9, 128.7, 128.2, 127.7, 127.2 (2x), 127.1, 127.0, 124.7, 122.5, 117.3, 78.5, 65.6, 53.1, 49.8 ppm; IR (KBr) $\tilde{\nu}$: 3434, 2919, 2850, 1704, 1606, 1468, 1383, 1302, 1106, 965, 759, 694 cm^{-1}; EIMS *m/z* (%): 348.9 (93) $[M]^+$, 283.0 (63), 255.0 (100); $[\alpha]_D^{RT}$ = −135 (*c* 6.6, MeOH); HPLC conditions: AD-H column, *n*-hexane/2-propanol = 90/10, flow rate = 0.6 mL·min^{-1}, major enantiomer: t_R = 19.41 min; minor enantiomer: t_R = 38.86 min.

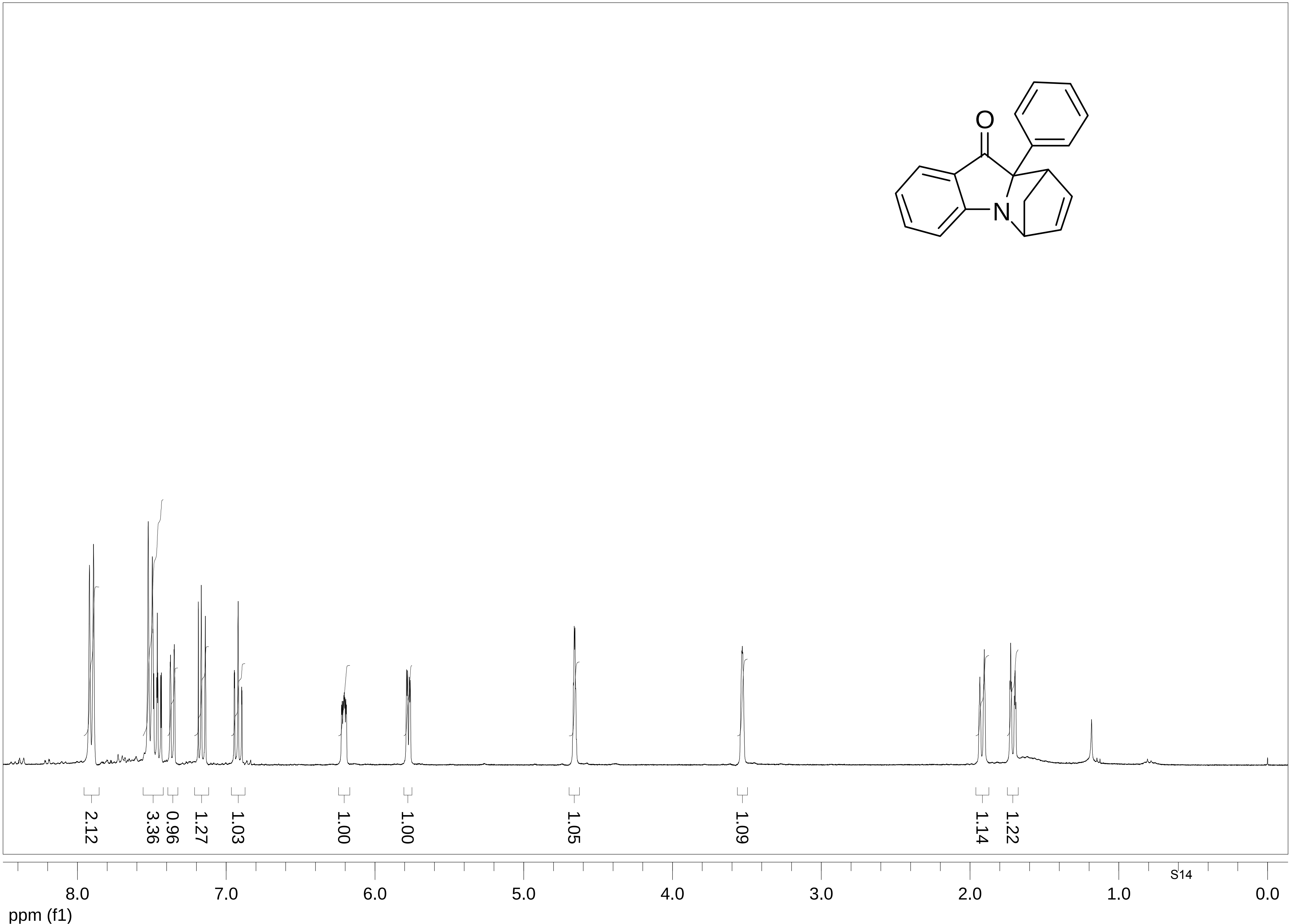
O
N
2.12
3.36
0.96
1.27
1.03
1.00
1.00
1.05
1.09
1.14
1.22
8.0
7.0
6.0
5.0
4.0
3.0
2.0
1.0
0.0
ppm (f1)

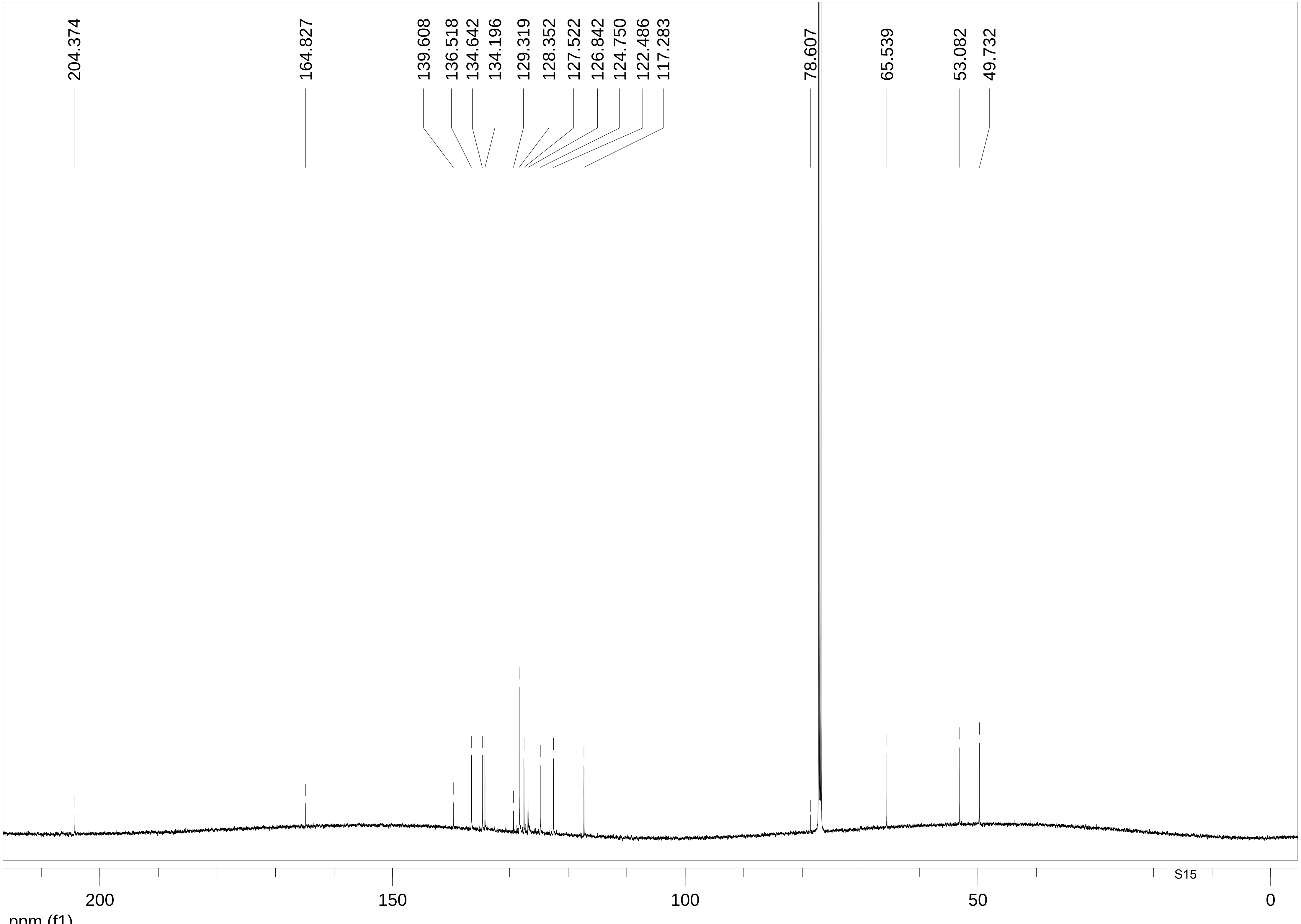
204.374
164.827
139.608
136.518
134.642
134.196
129.319
128.352
127.522
126.842
124.750
122.486
117.283
78.607
65.539
53.082
49.732
200
150
100
50
0
ppm (f1)

Chromatogram : SR_586rac_ADH_9010_flow06_acq605

Data file: SR_586rac_ADH_9010_flow06_acq605.DATA
Method: HPLC2_ADH_9010_flow06_acq60
Date: 11.11.2011 15:20:29

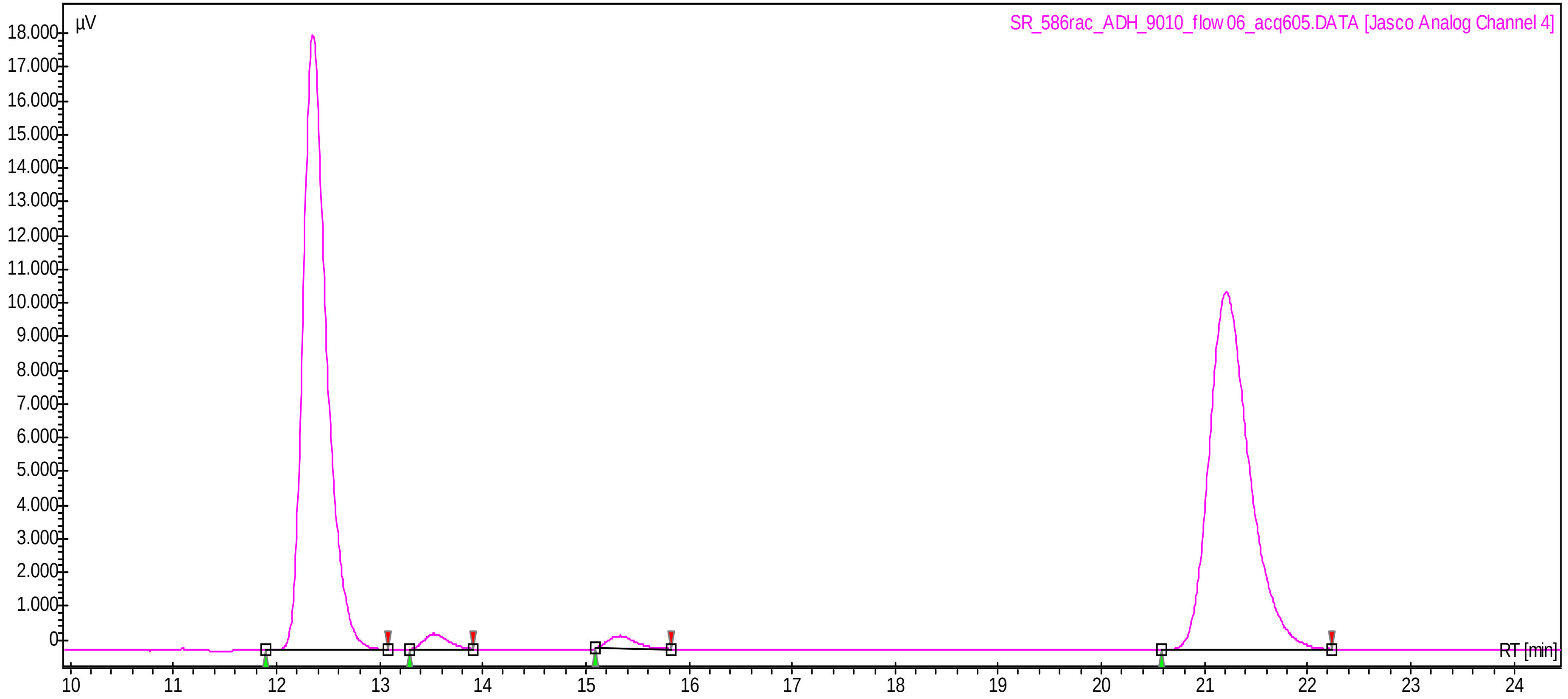

SR_586rac_ADH_9010_flow06_acq605.DATA [Jasco Analog Channel 4]

Index	Start	Time	End	Area %
	[Min]	[Min]	[Min]	[%]
1	11,892	12,350	13,081	48,762
3	13,288	13,525	13,909	1,224
4	15,098	15,333	15,822	1,157
2	20,578	21,217	22,233	48,857
Total				100,000

Chromatogram : SR_586_c_ADH_9010_flow06_acq302

Data file: SR_586_c_ADH_9010_flow06_acq302.DATA
Method: HPLC2_ADH_9010_flow06_acq30
Date: 15.11.2011 14:33:00

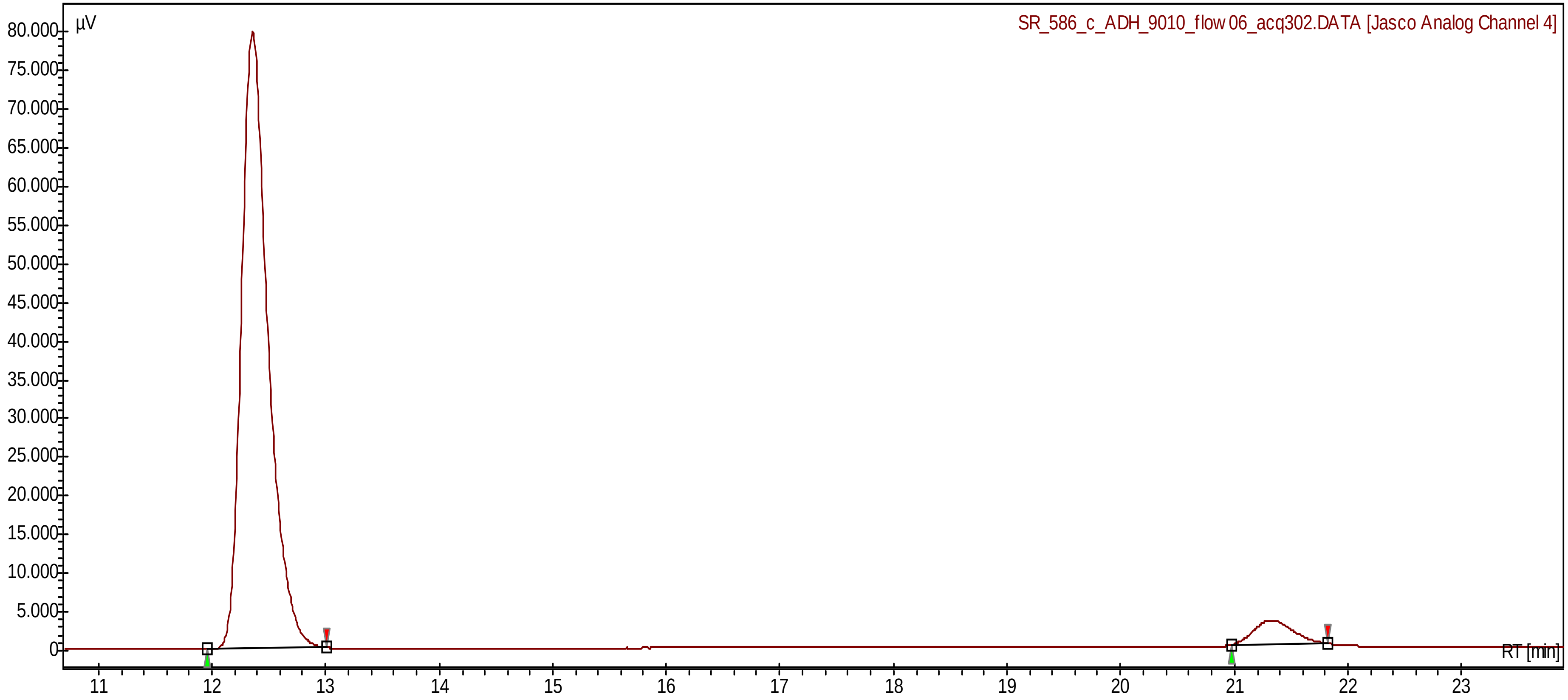

SR_586_c_ADH_9010_flow06_acq302.DATA [Jasco Analog Channel 4]

Index	Start	Time	End	Area %
	[Min]	[Min]	[Min]	[%]
1	11,963	12,358	13,017	94,668
2	20,981	21,325	21,818	5,332
Total				100,000

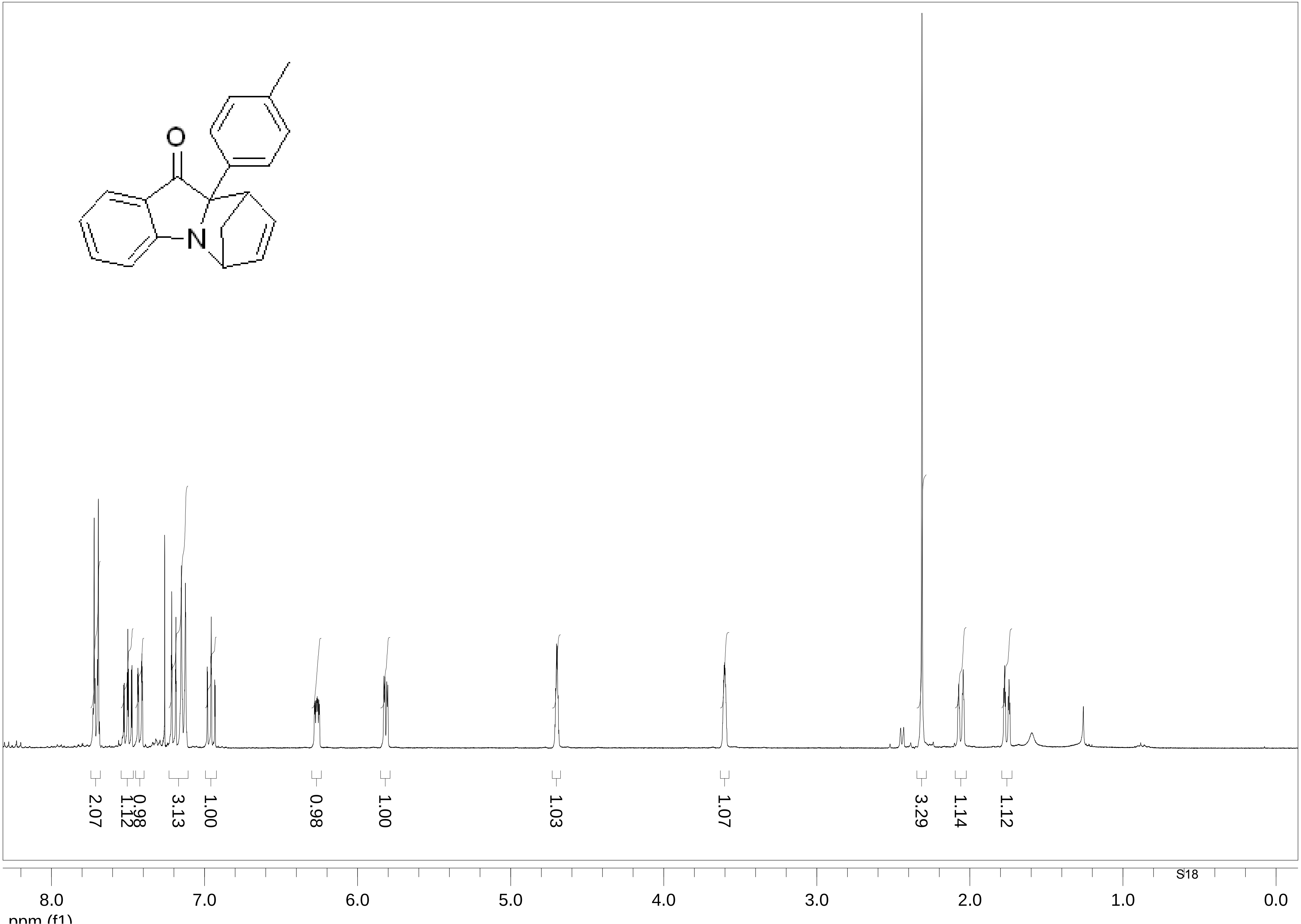
O
N
2.07
1.12
0.98
3.13
1.00
0.98
1.00
1.03
1.07
3.29
1.14
1.12
8.0
7.0
6.0
5.0
4.0
3.0
2.0
1.0
0.0
ppm (f1)

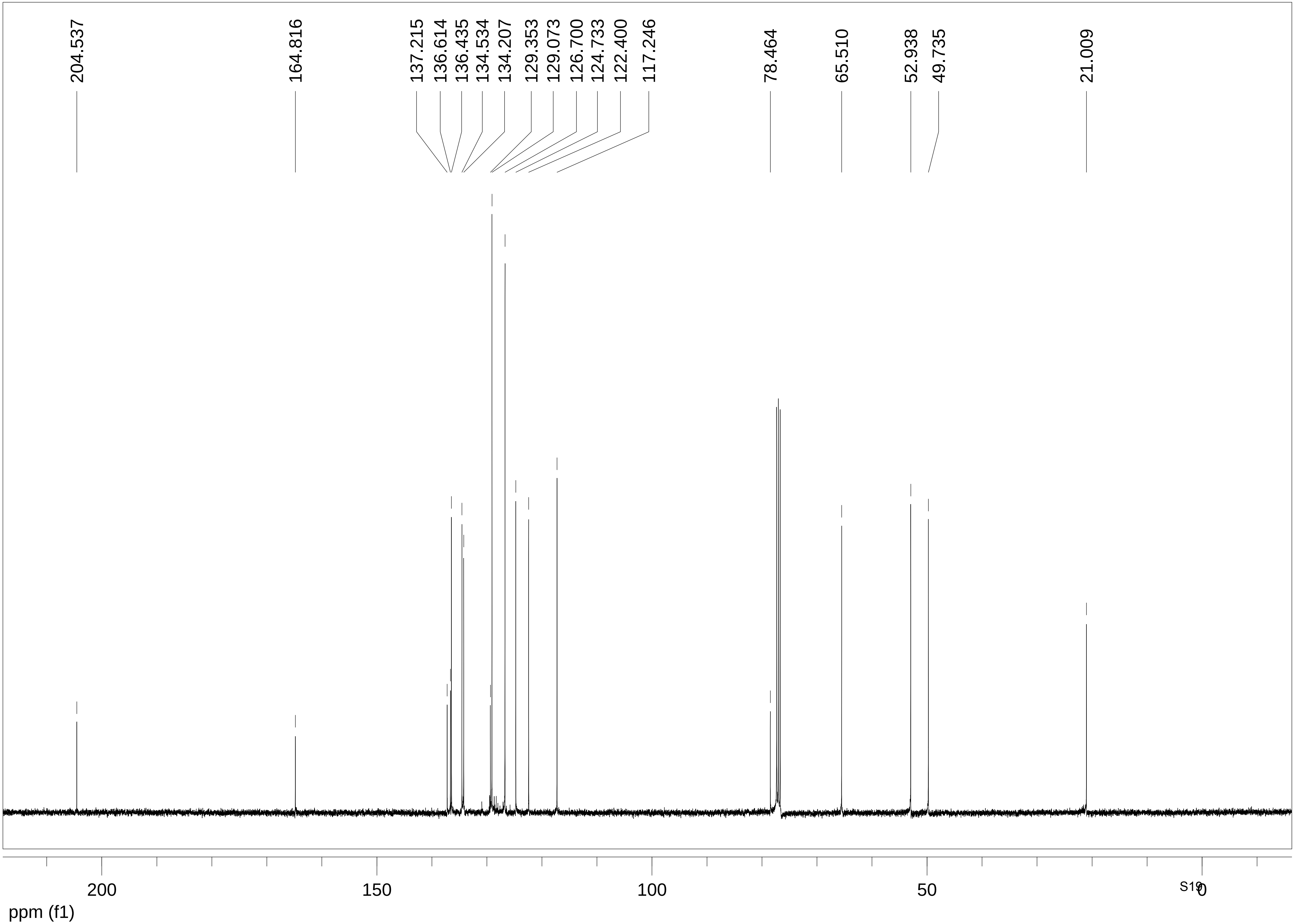
204.537
164.816
137.215
136.614
136.435
134.534
134.207
129.353
129.073
126.700
124.733
122.400
117.246
78.464
65.510
52.938
49.735
21.009
200
150
100
50
0
ppm (f1)

Chromatogram : SR_579rac_ADH_9010_flow06_acq4015

Data file: SR_579rac_ADH_9010_flow06_acq4015.DATA
Method: HPLC2_ADH_9010_flow06_acq40
Date: 16.11.2011 22:38:00

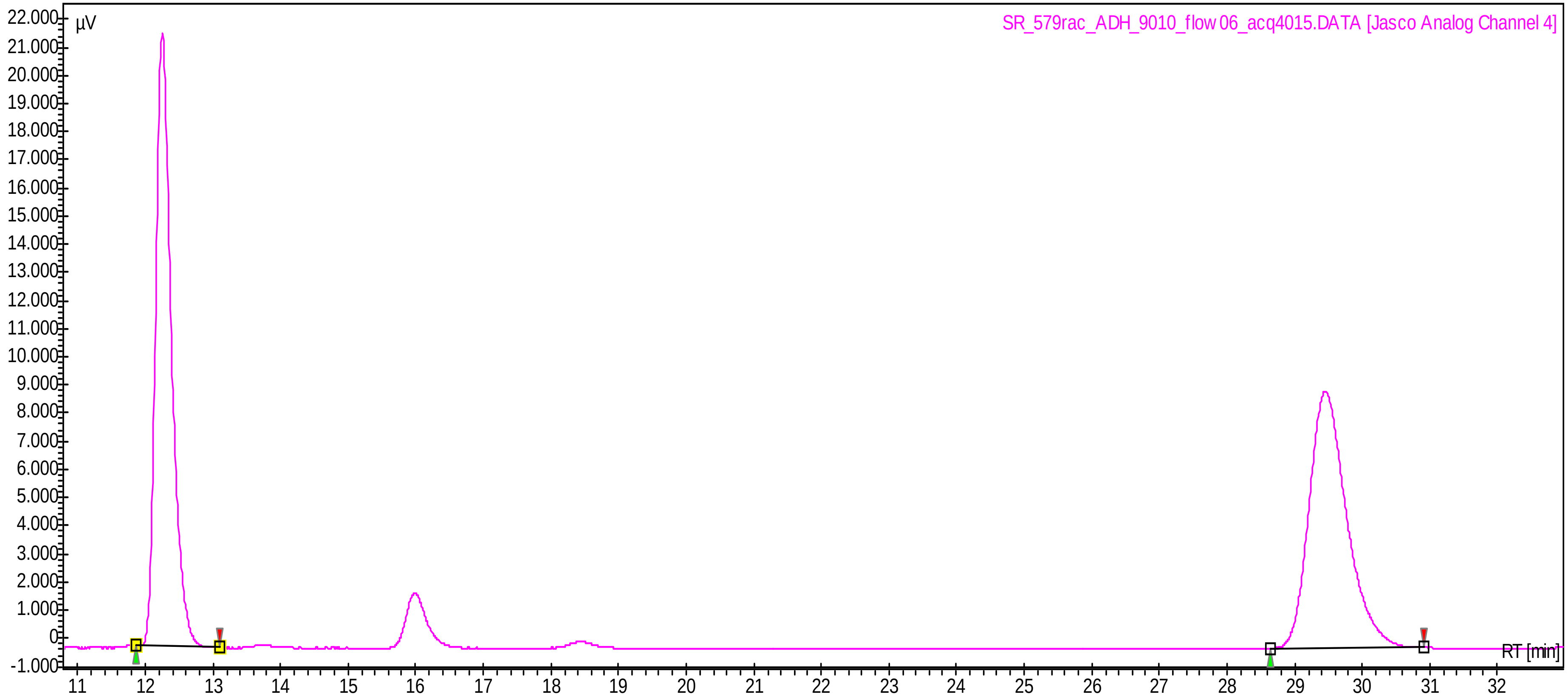

SR_579rac_ADH_9010_flow06_acq4015.DATA [Jasco Analog Channel 4]

Index	Start	Time	End	Area %
	[Min]	[Min]	[Min]	[%]
1	11,860	12,250	13,099	50,000
2	28,636	29,458	30,909	50,000
Total				100,000

Chromatogram : SR_579.4.9_ADH_9010_flow06_acq402

Data file: SR_579.4.9_ADH_9010_flow06_acq402.DATA
Method: HPLC2_ADH_9010_flow06_acq40
Date: 11.11.2011 12:21:36

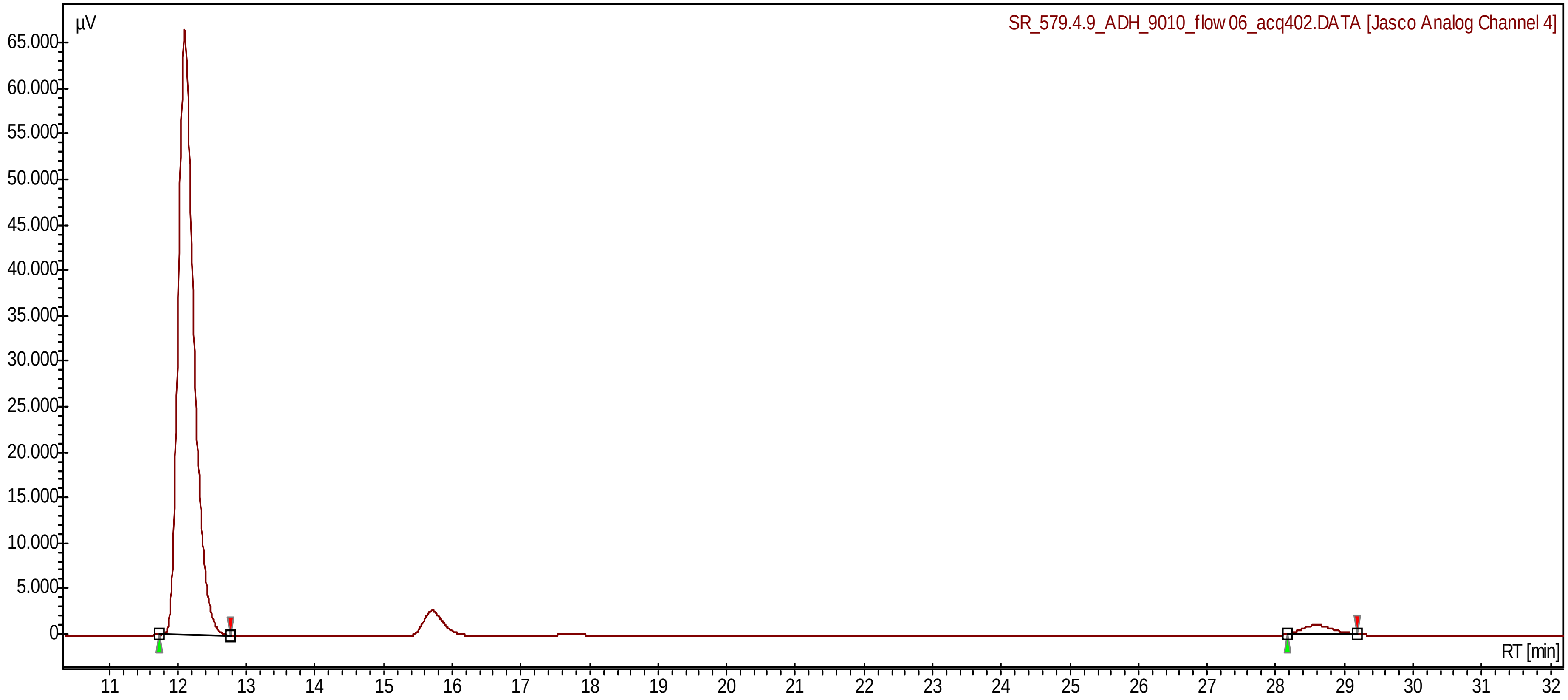

SR_579.4.9_ADH_9010_flow06_acq402.DATA [Jasco Analog Channel 4]

Index	Start	Time	End	Area %
	[Min]	[Min]	[Min]	[%]
1	11,736	12,100	12,769	97,190
2	28,170	28,575	29,187	2,810
Total				100,000

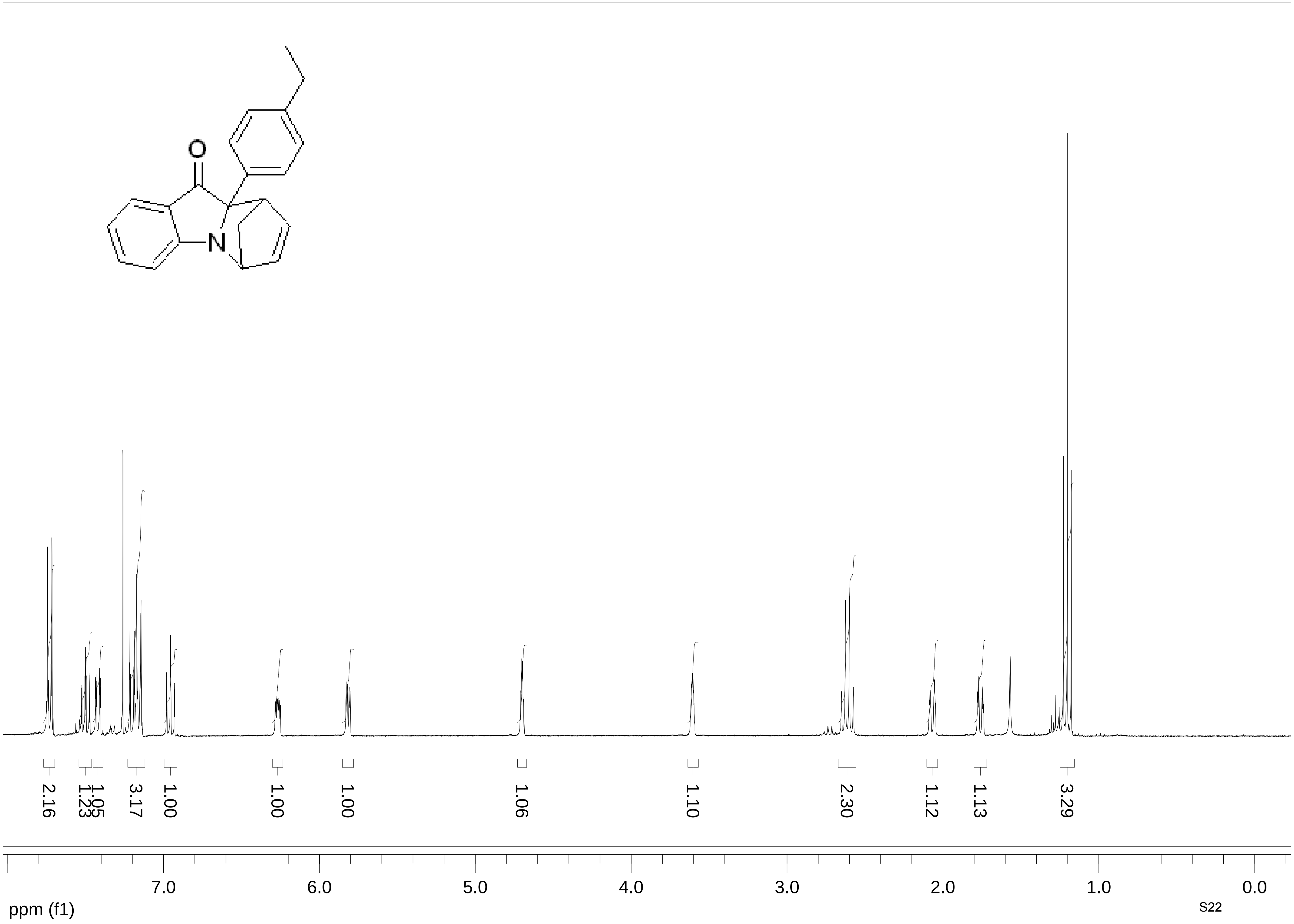
O
N
2.16
1.23
1.05
3.17
1.00
1.00
1.00
1.06
1.10
2.30
1.12
1.13
3.29
7.0
6.0
5.0
4.0
3.0
2.0
1.0
0.0
ppm (f1)

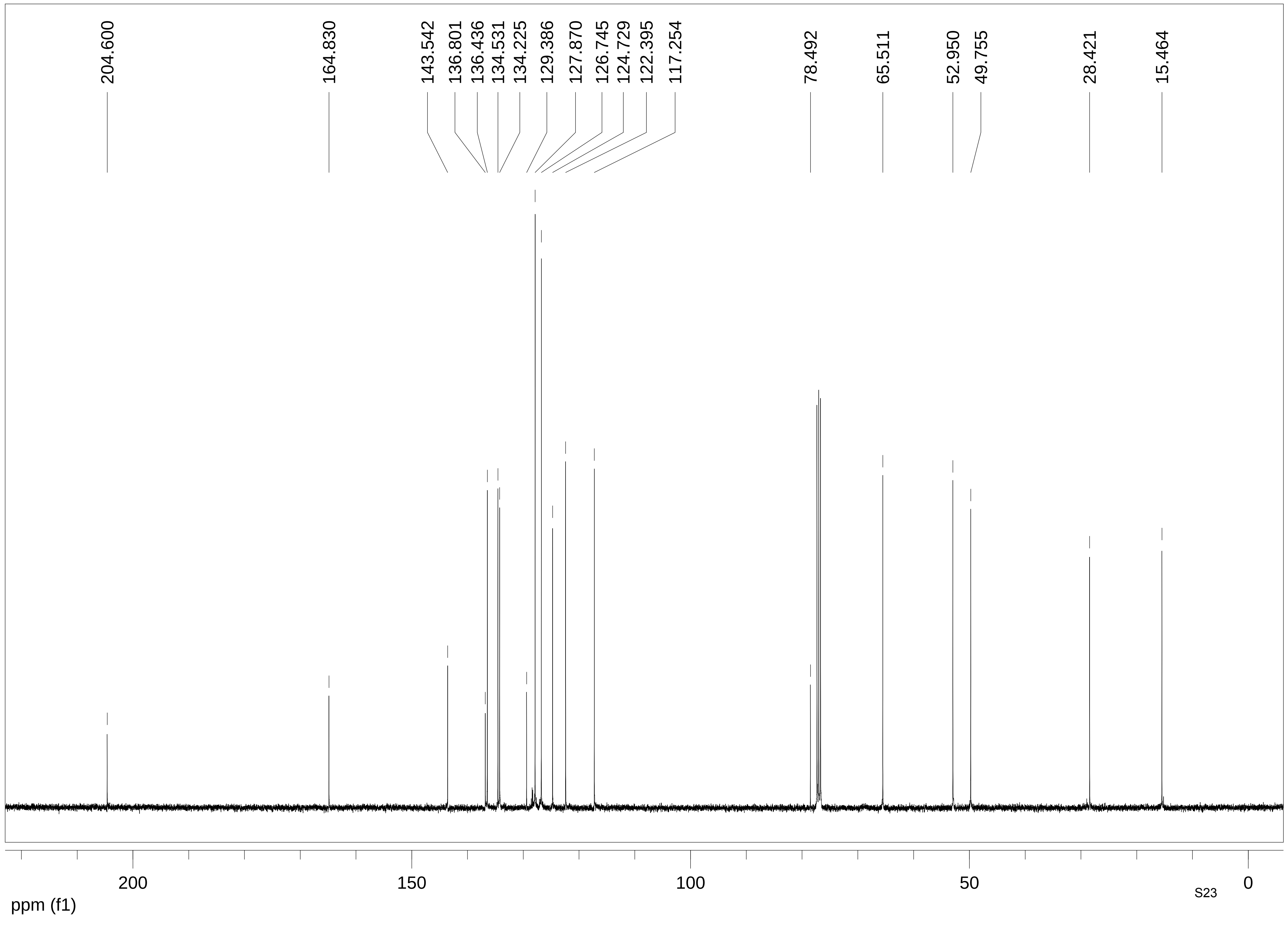
204.600
164.830
143.542
136.801
136.436
134.531
134.225
129.386
127.870
126.745
124.729
122.395
117.254
78.492
65.511
52.950
49.755
28.421
15.464
200
150
100
50
0
ppm (f1)

Chromatogram : SR_587rac_ADH_9010_flow06_acq6016

Data file: SR_587rac_ADH_9010_flow06_acq6016.DATA
Method: HPLC2_ADH_9010_flow06_acq50
Date: 16.11.2011 23:20:36

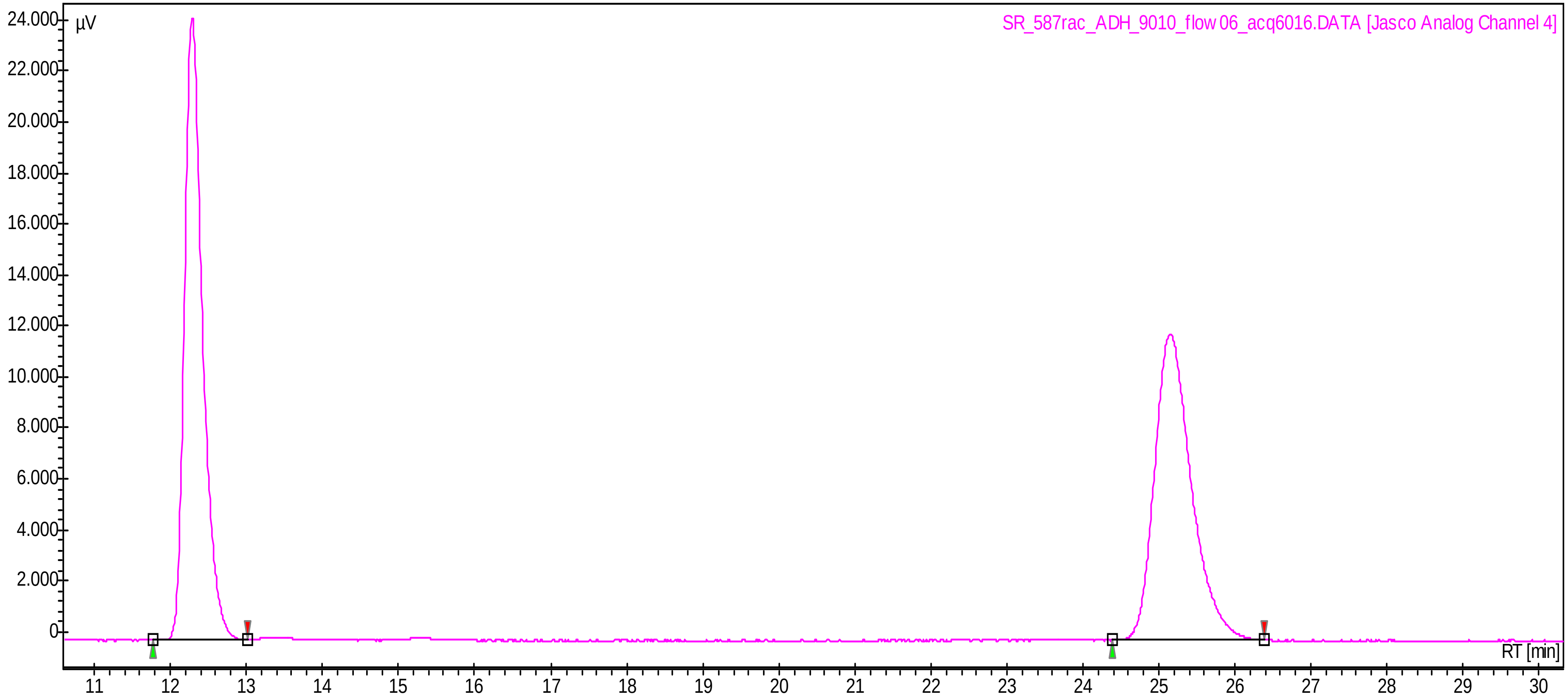

SR_587rac_ADH_9010_flow06_acq6016.DATA [Jasco Analog Channel 4]

Index	Start	Time	End	Area %
	[Min]	[Min]	[Min]	[%]
1	11,777	12,283	13,017	49,937
2	24,380	25,142	26,395	50,063
Total				100,000

Chromatogram : SR_587.4_c_ADH_9010_flow06_acq402

Data file: SR_587.4_c_ADH_9010_flow06_acq402.DATA
Method: HPLC2_ADH_9010_flow06_acq40
Date: 22.11.2011 11:48:23

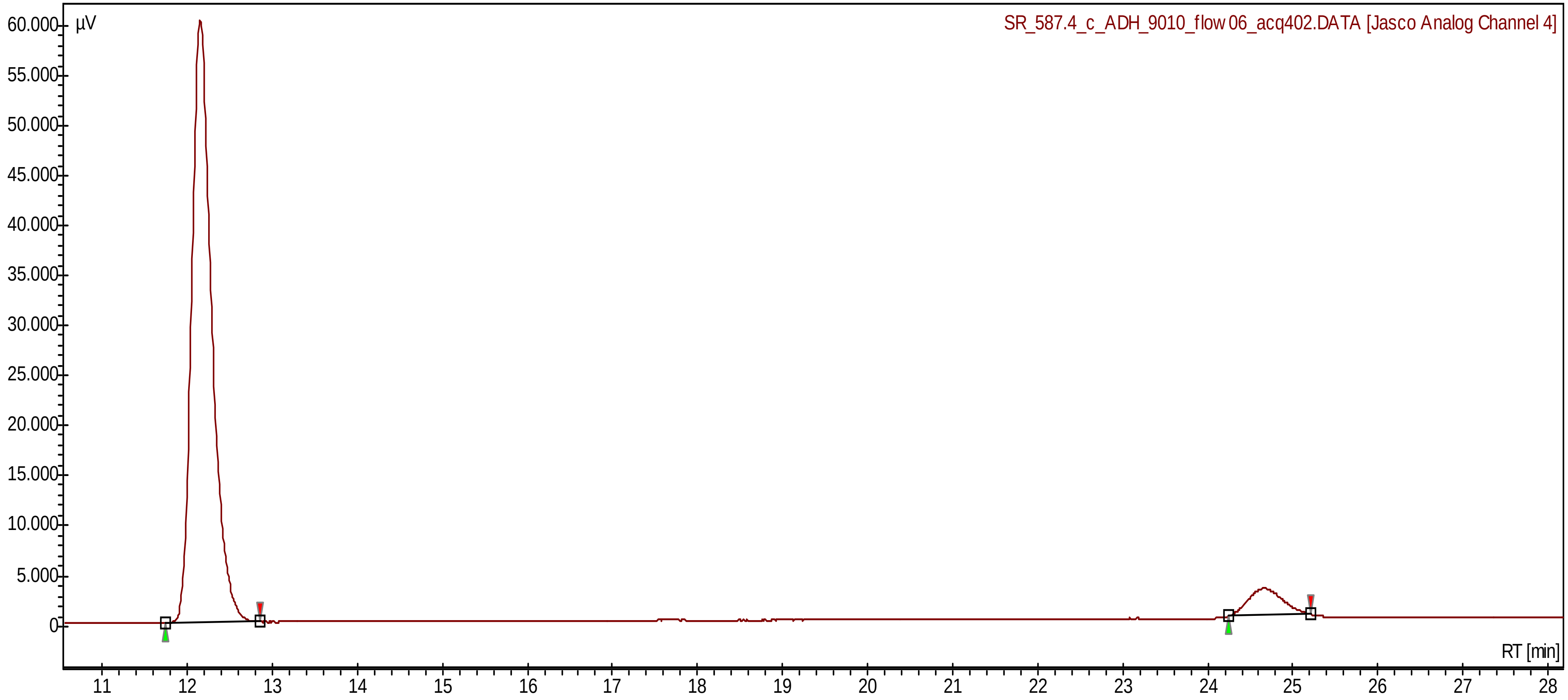

SR_587.4_c_ADH_9010_flow06_acq402.DATA [Jasco Analog Channel 4]

Index	Start	Time	End	Area %
	[Min]	[Min]	[Min]	[%]
1	11,736	12,150	12,851	92,837
2	24,241	24,650	25,215	7,163
Total				100,000

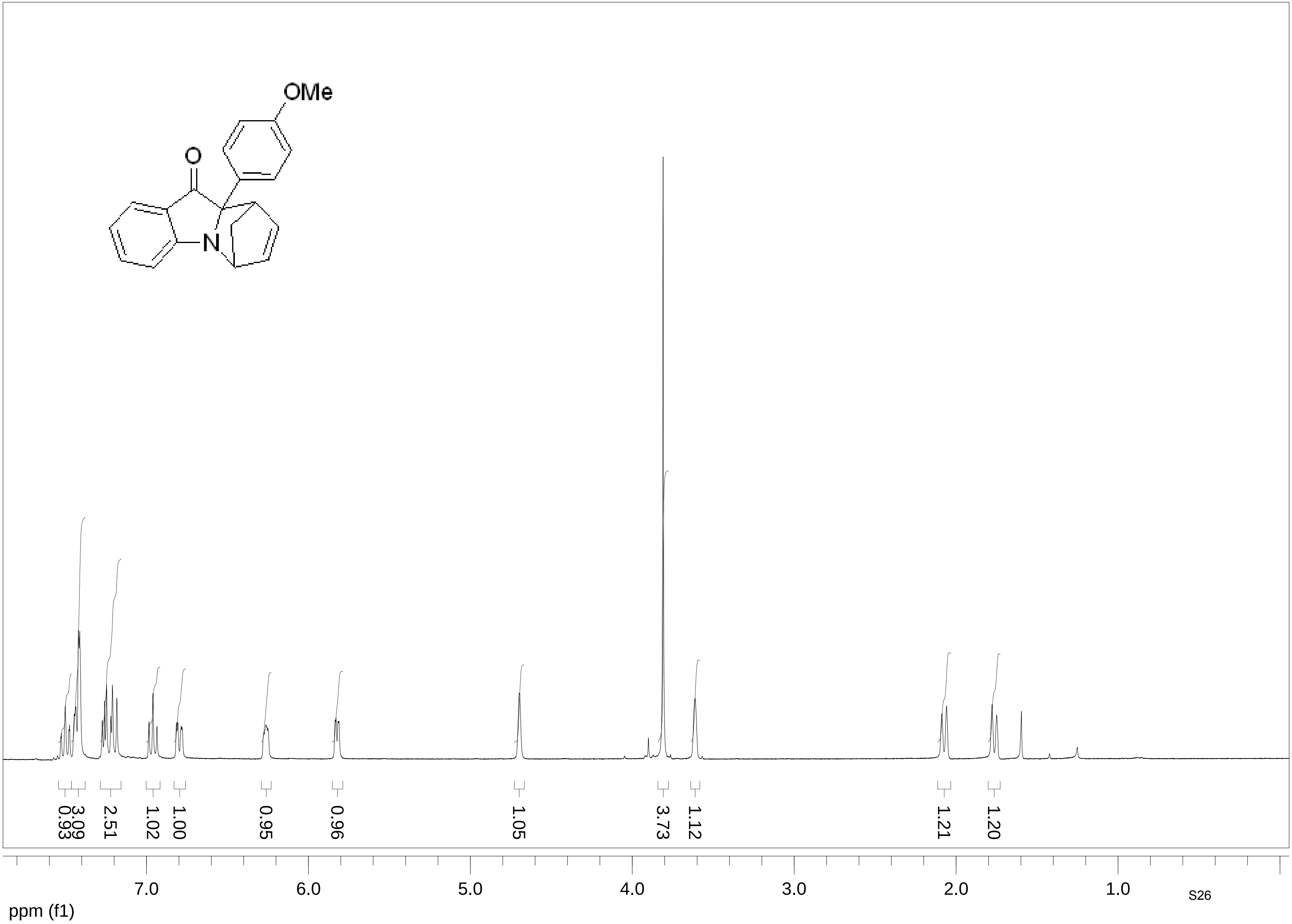
OMe
O
N
0.93
3.09
2.51
1.02
1.00
0.95
0.96
1.05
3.73
1.12
1.21
1.20
7.0
6.0
5.0
4.0
3.0
2.0
1.0
ppm (f1)

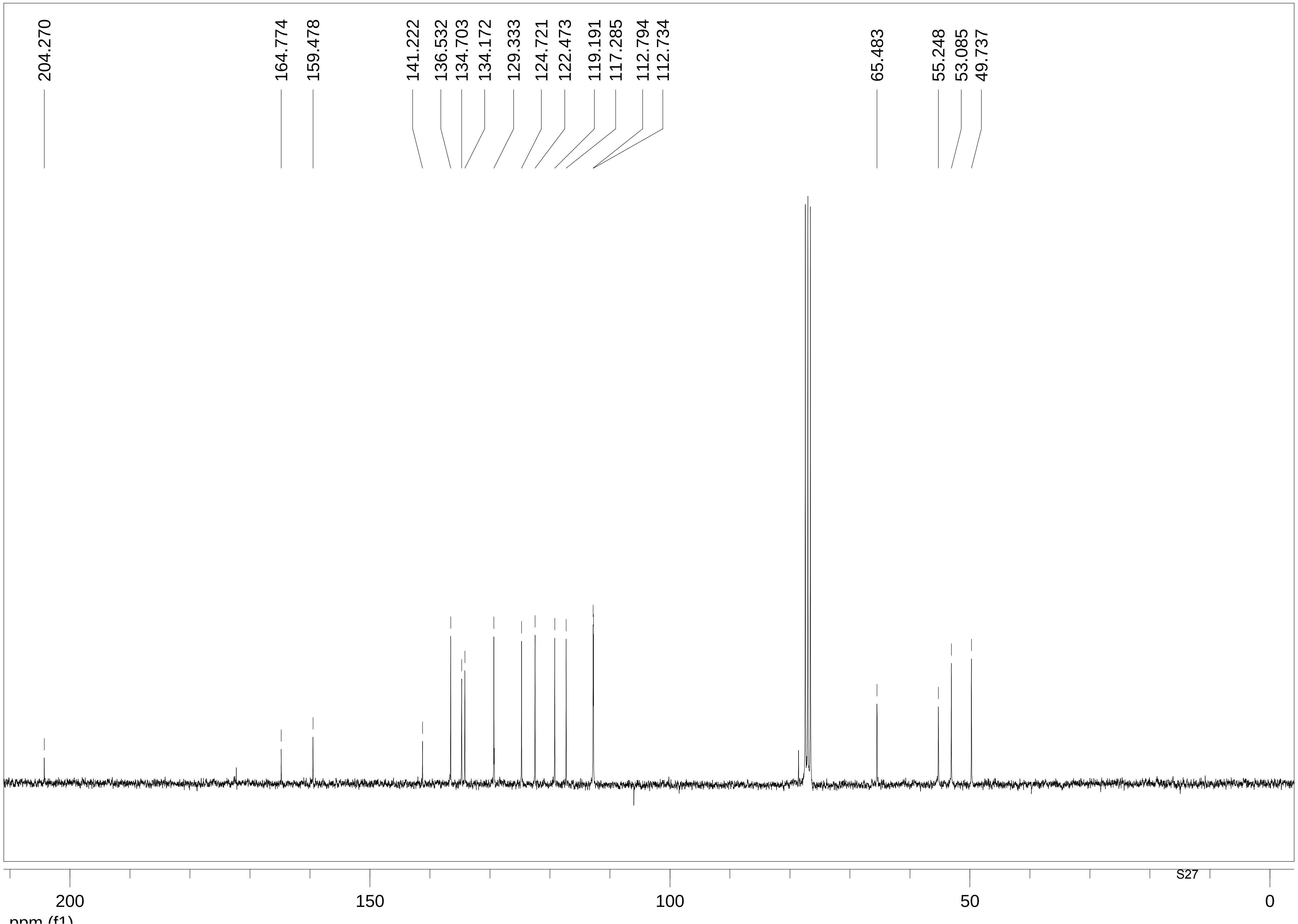
204.270
164.774
159.478
141.222
136.532
134.703
134.172
129.333
124.721
122.473
119.191
117.285
112.794
112.734
65.483
55.248
53.085
49.737
200
150
100
50
0
ppm (f1)

Chromatogram : SR_588_rac_ADH_9010_flow06_acq6010

Data file: SR_588_rac_ADH_9010_flow06_acq6010.DATA
Method: HPLC2_ADH_9010_flow06_acq60
Date: 17.11.2011 17:44:05

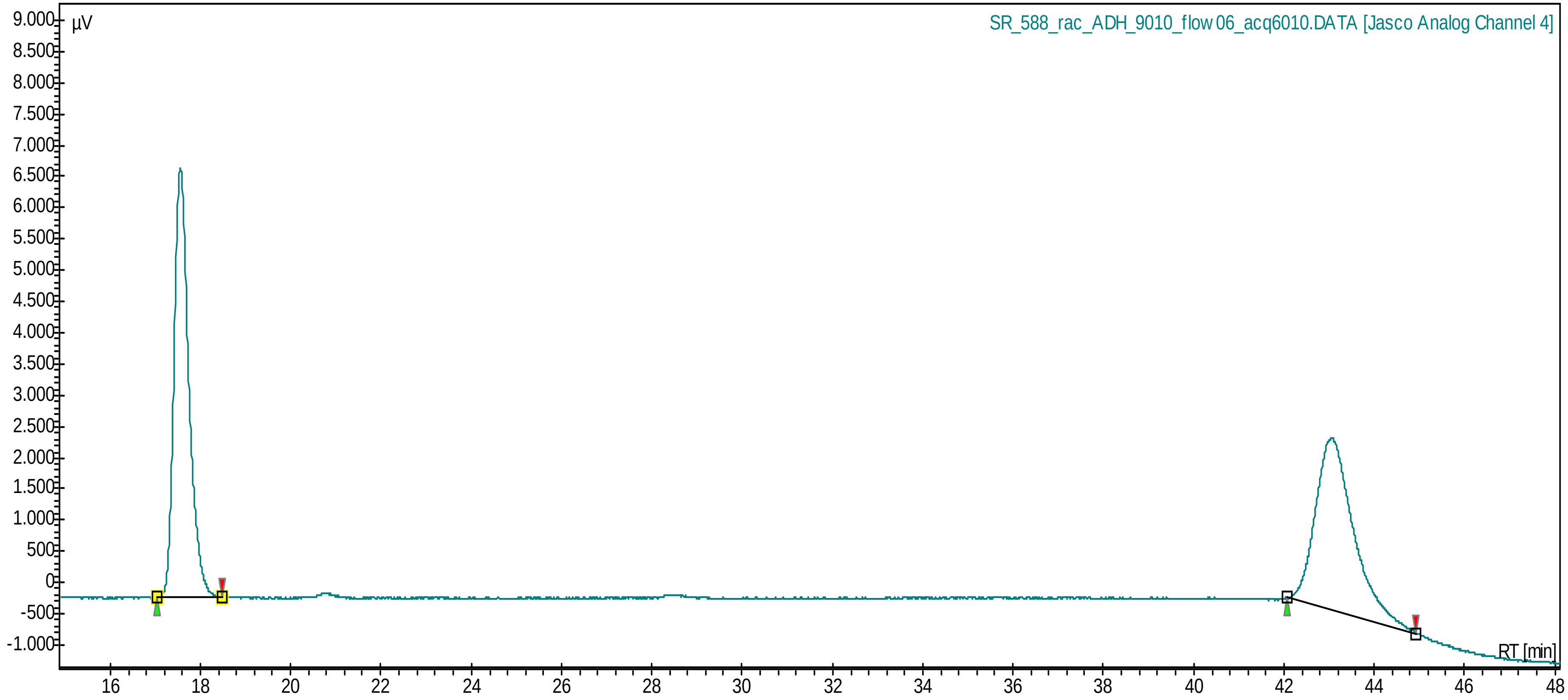

SR_588_rac_ADH_9010_flow06_acq6010.DATA [Jasco Analog Channel 4]

Index	Start	Time	End	Area %
	[Min]	[Min]	[Min]	[%]
1	17,045	17,558	18,471	49,233
2	42,087	43,075	44,938	50,767
Total				100,000

Chromatogram : SR_588_c_ADH_9010_flow06_acq604

Data file: SR_588_c_ADH_9010_flow06_acq604.DATA
Method: HPLC2_ADH_9010_flow06_acq60
Date: 19.11.2011 12:26:40

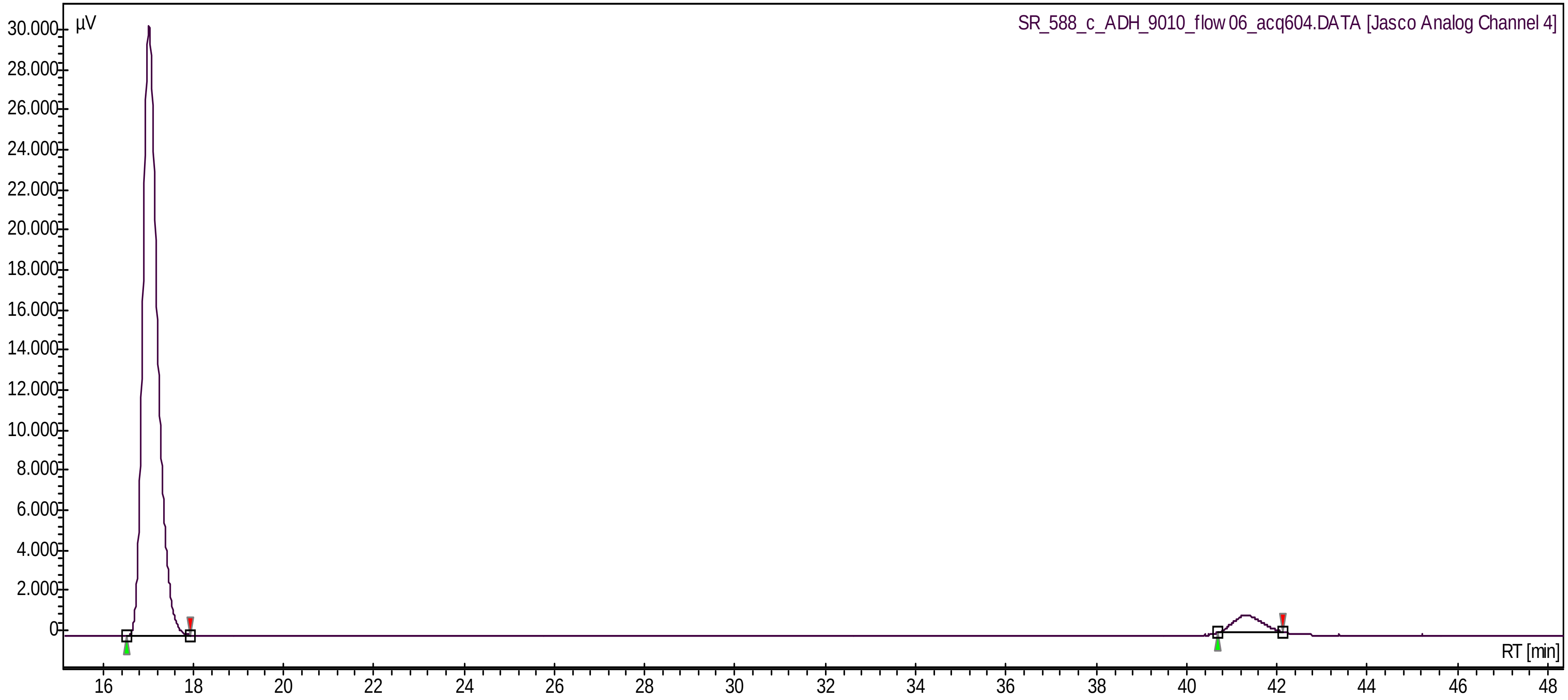

SR_588_c_ADH_9010_flow06_acq604.DATA [Jasco Analog Channel 4]

Index	Start	Time	End	Area %
	[Min]	[Min]	[Min]	[%]
1	16,531	17,025	17,938	94,921
2	40,693	41,325	42,135	5,079
Total				100,000

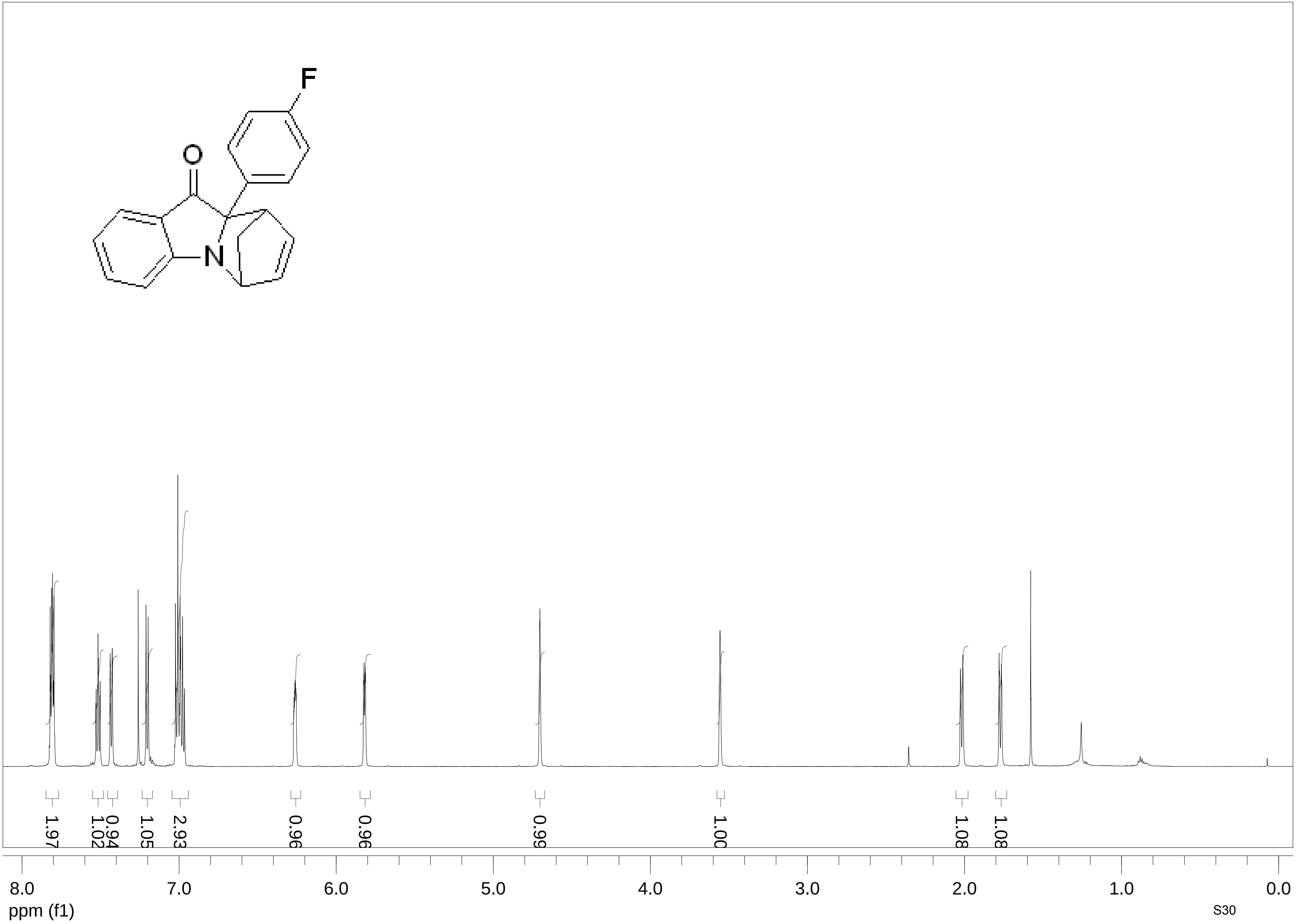
F
O
N
1.97
1.02
0.94
1.05
2.93
0.96
0.96
0.99
1.00
1.08
1.08
8.0
7.0
6.0
5.0
4.0
3.0
2.0
1.0
0.0
ppm (f1)

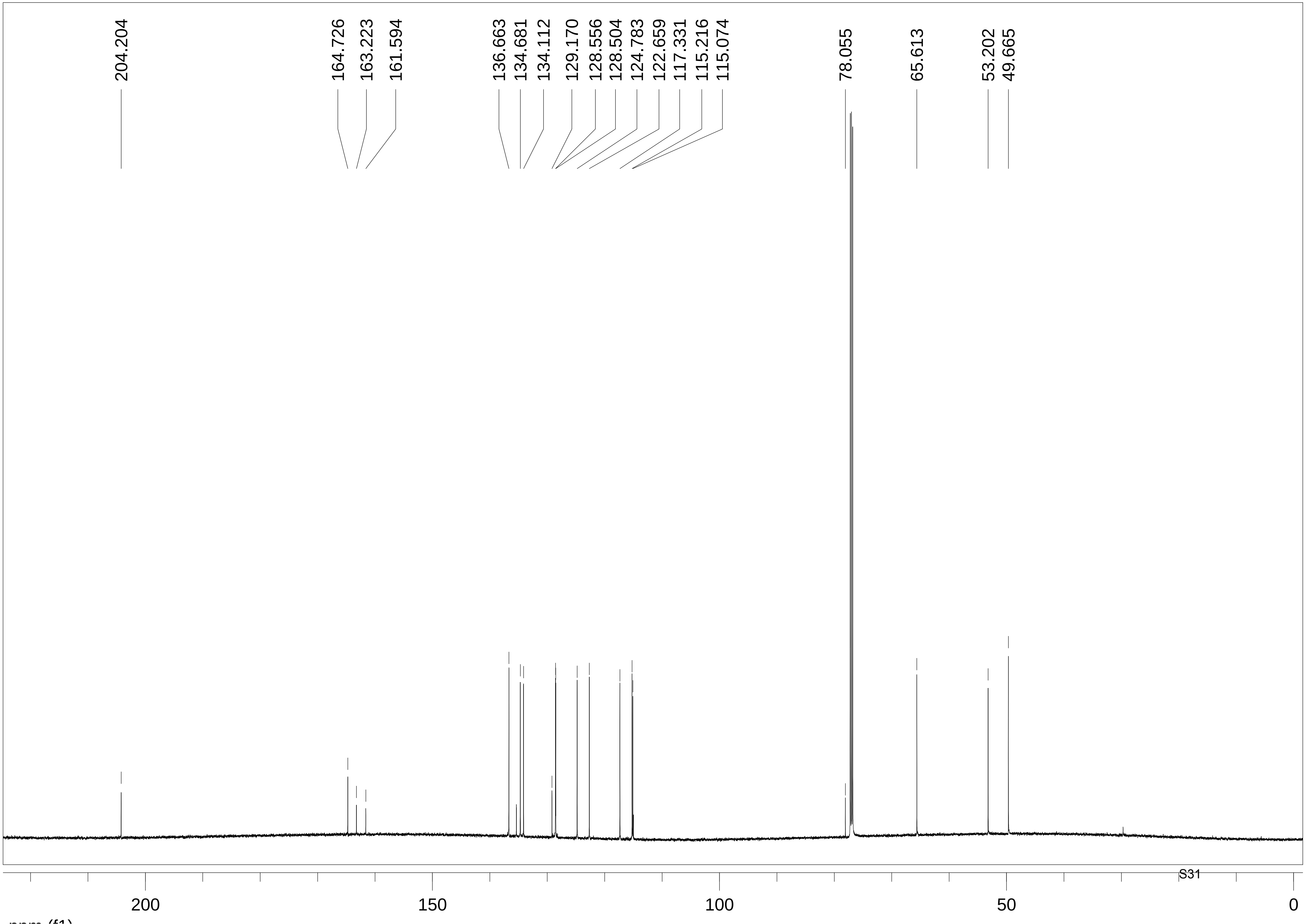
204.204
164.726
163.223
161.594
136.663
134.681
134.112
129.170
128.556
128.504
124.783
122.659
117.331
115.216
115.074
78.055
65.613
53.202
49.665
200
150
100
50
0
ppm (f1)

Chromatogram : SR_591rac_ADH_9010_flow06_acq607

Data file: SR_591rac_ADH_9010_flow06_acq607.DATA
Method: HPLC2_ADH_9010_flow06_acq60
Date: 11.11.2011 17:15:35

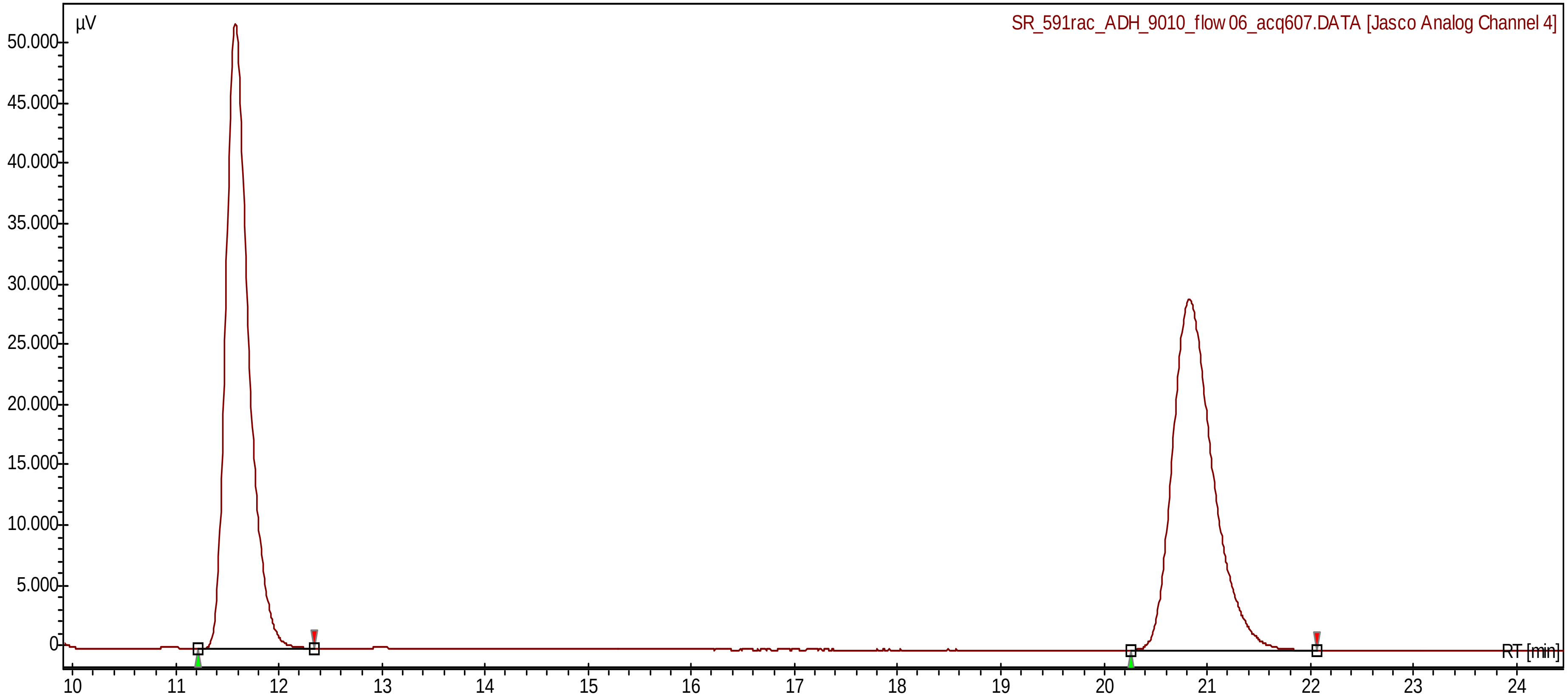

SR_591rac_ADH_9010_flow06_acq607.DATA [Jasco Analog Channel 4]

Index	Start	Time	End	Area %
	[Min]	[Min]	[Min]	[%]
1	11,219	11,575	12,335	49,991
2	20,269	20,825	22,066	50,009
Total				100,000

Chromatogram : SR_591c_ADH_9010_flow06_acq403

Data file: SR_591c_ADH_9010_flow06_acq403.DATA
Method: HPLC2_ADH_9010_flow06_acq40
Date: 17.11.2011 11:55:23

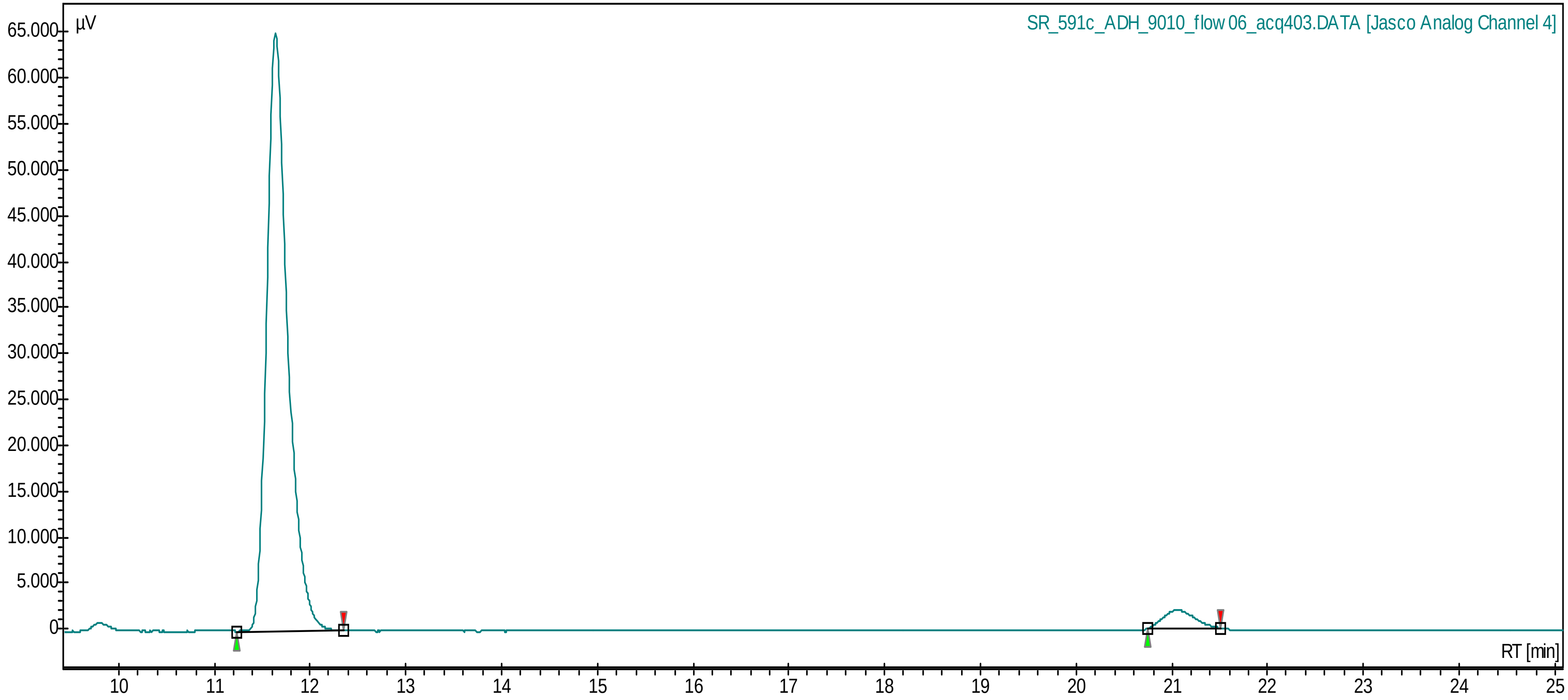

SR_591c_ADH_9010_flow06_acq403.DATA [Jasco Analog Channel 4]

Index	Start	Time	End	Area %
	[Min]	[Min]	[Min]	[%]
1	11,240	11,642	12,355	95,665
2	20,747	21,058	21,507	4,335
Total				100,000

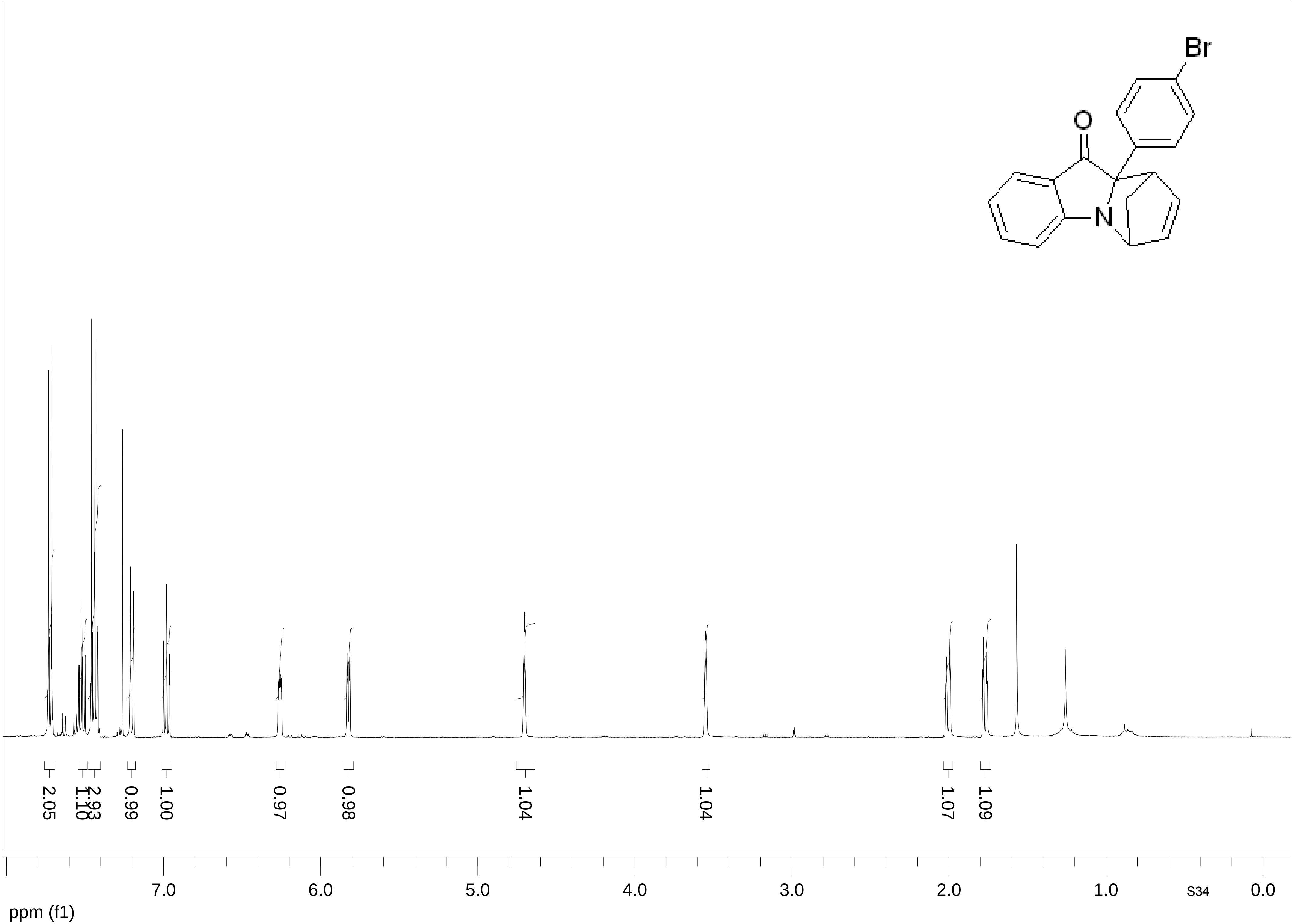
Br
O
N
2.05
1.10
2.93
0.99
1.00
0.97
0.98
1.04
1.04
1.07
1.09
7.0
6.0
5.0
4.0
3.0
2.0
1.0
0.0
ppm (f1)

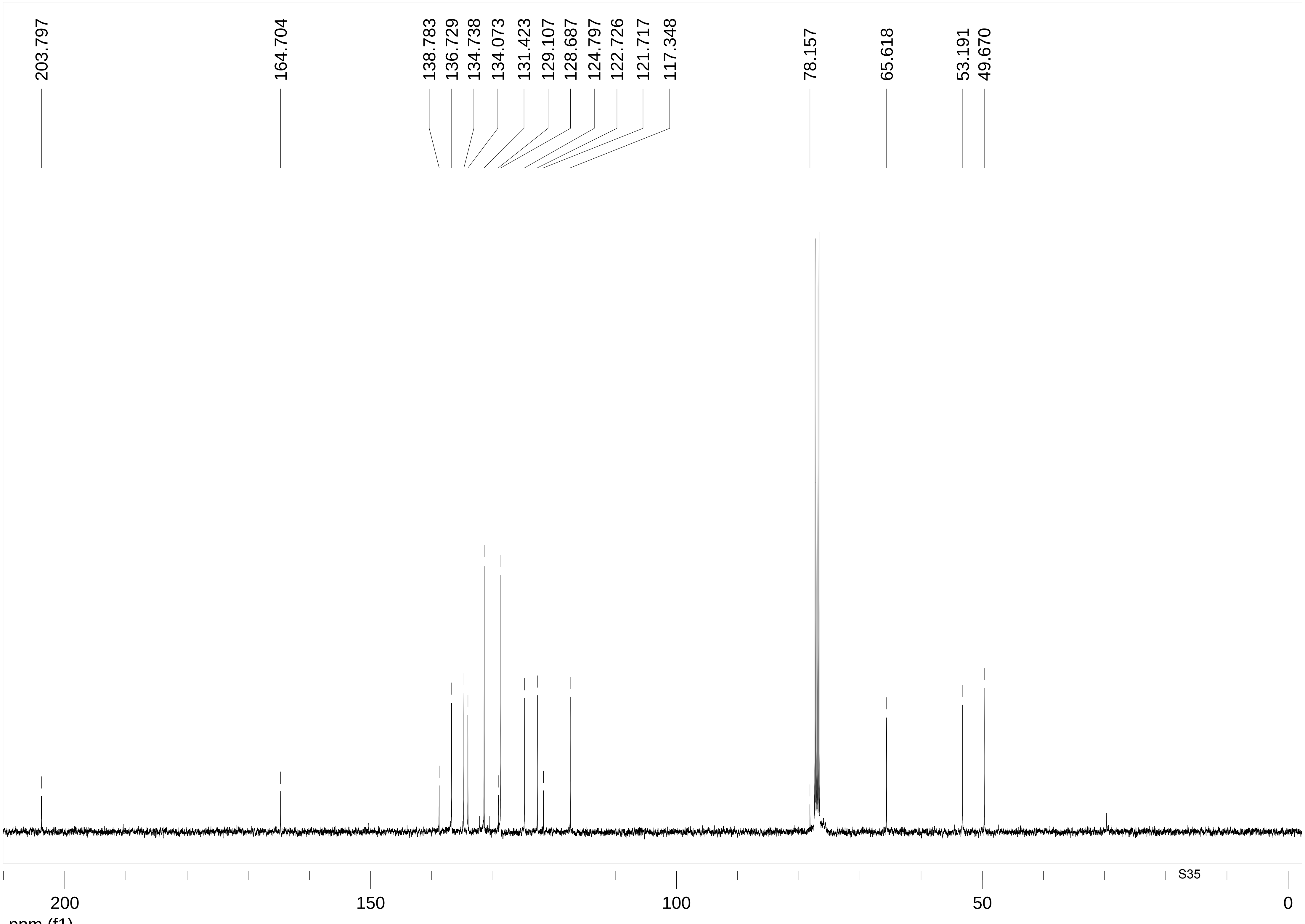
203.797
164.704
138.783
136.729
134.738
134.073
131.423
129.107
128.687
124.797
122.726
121.717
117.348
78.157
65.618
53.191
49.670
200
150
100
50
0
ppm (f1)

Chromatogram : SR_589rac_ADH_9010_flow06_acq404

Data file: SR_589rac_ADH_9010_flow06_acq404.DATA
Method: HPLC2_ADH_9010_flow06_acq40
Date: 17.11.2011 12:37:59

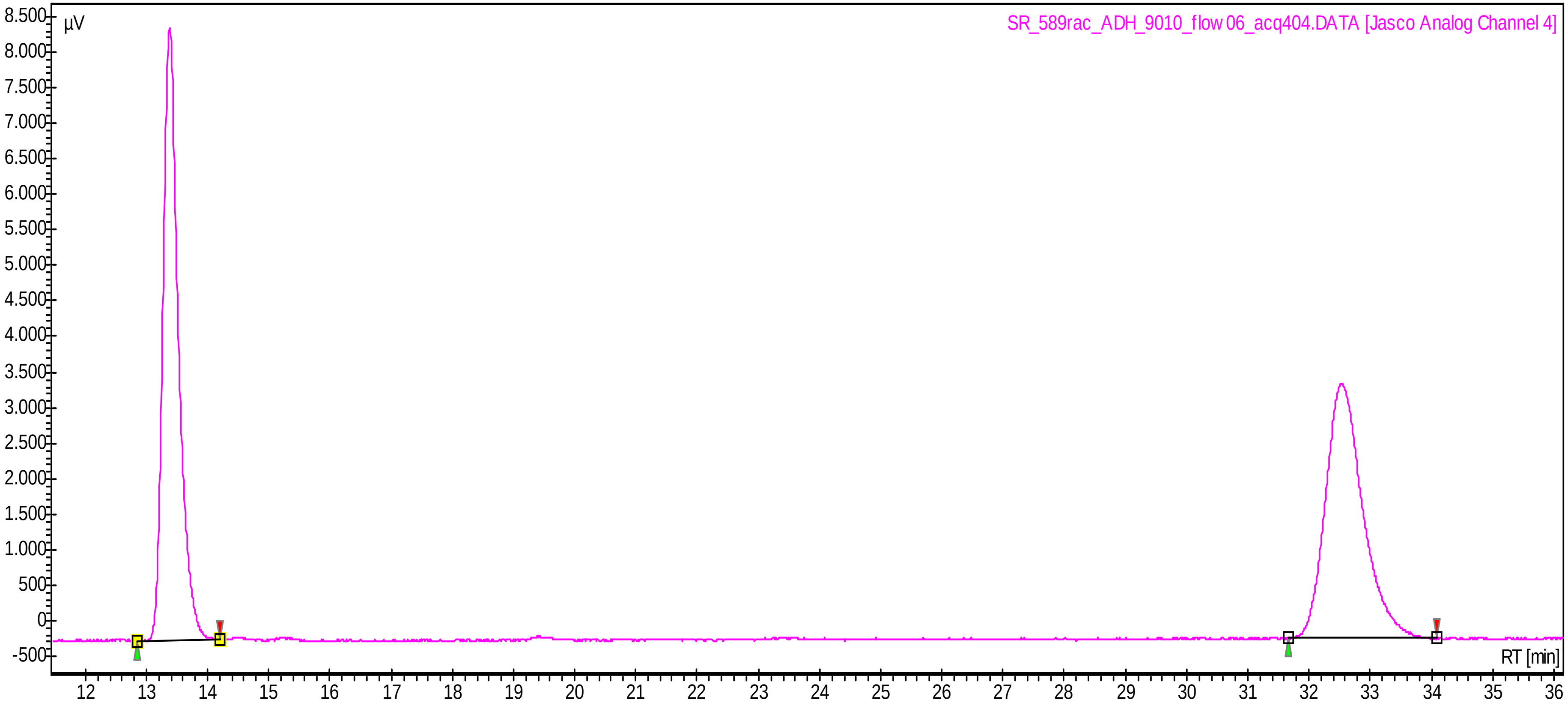

SR_589rac_ADH_9010_flow06_acq404.DATA [Jasco Analog Channel 4]

Index	Start	Time	End	Area %
	[Min]	[Min]	[Min]	[%]
1	12,851	13,375	14,215	50,142
2	31,653	32,542	34,091	49,858
Total				100,000

Chromatogram : SR_589.2_c_ADH_9010_flow06_acq403

Data file: SR_589.2_c_ADH_9010_flow06_acq403.DATA
Method: HPLC2_ADH_9010_flow06_acq40
Date: 19.11.2011 11:44:40

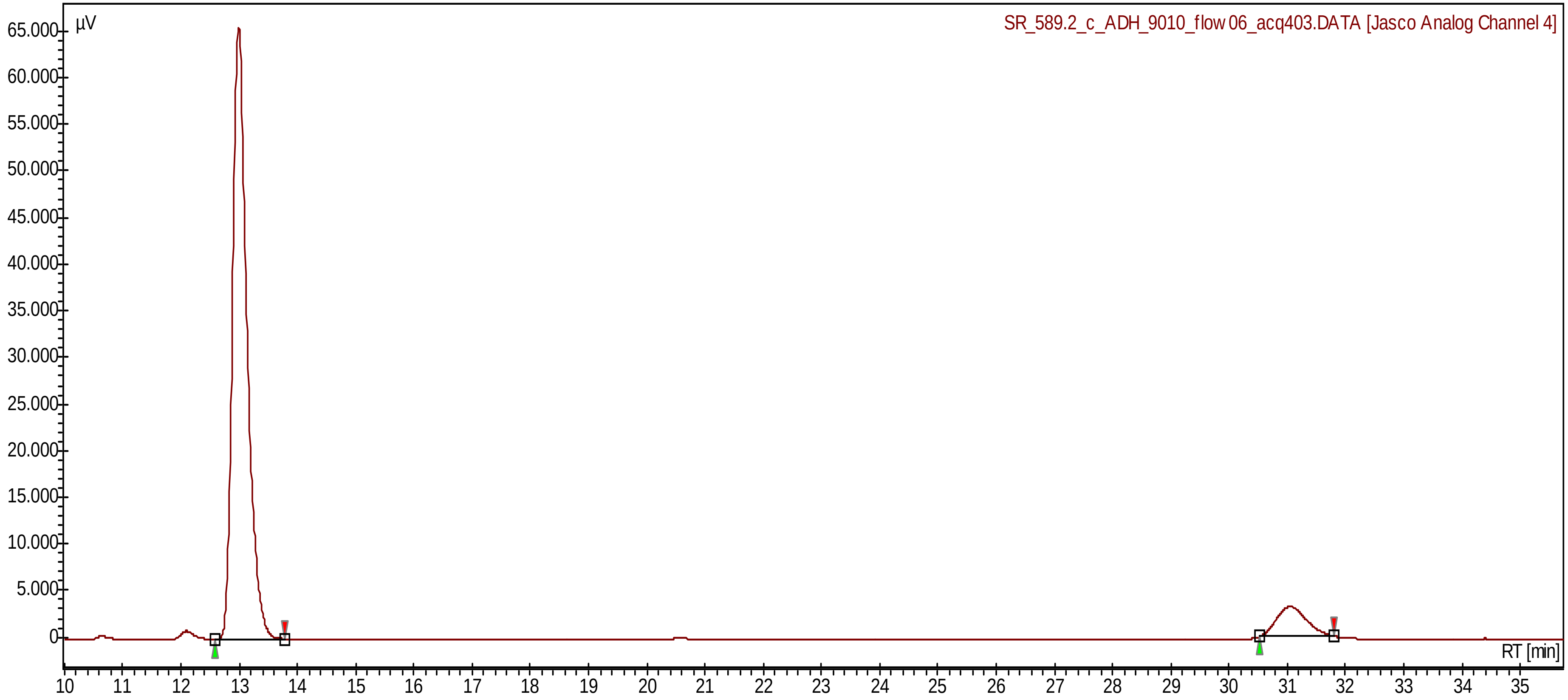

SR_589.2_c_ADH_9010_flow06_acq403.DATA [Jasco Analog Channel 4]

Index	Start	Time	End	Area %
	[Min]	[Min]	[Min]	[%]
1	12,595	12,992	13,781	90,838
2	30,523	31,050	31,801	9,162
Total				100,000

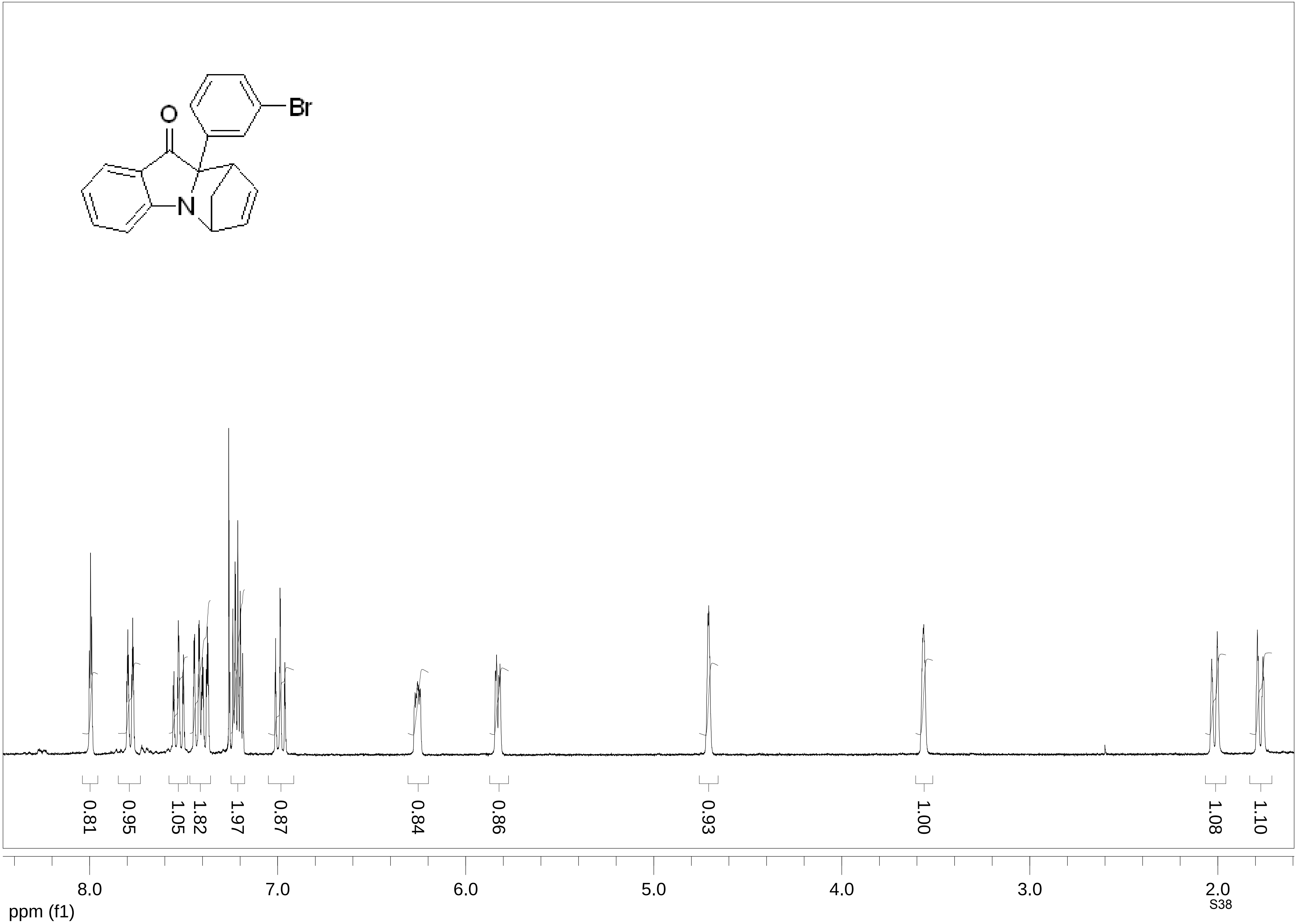

O
N
Br
0.81
0.95
1.05
1.82
1.97
0.87
0.84
0.86
0.93
1.00
1.08
1.10
8.0
7.0
6.0
5.0
4.0
3.0
2.0
ppm (f1)

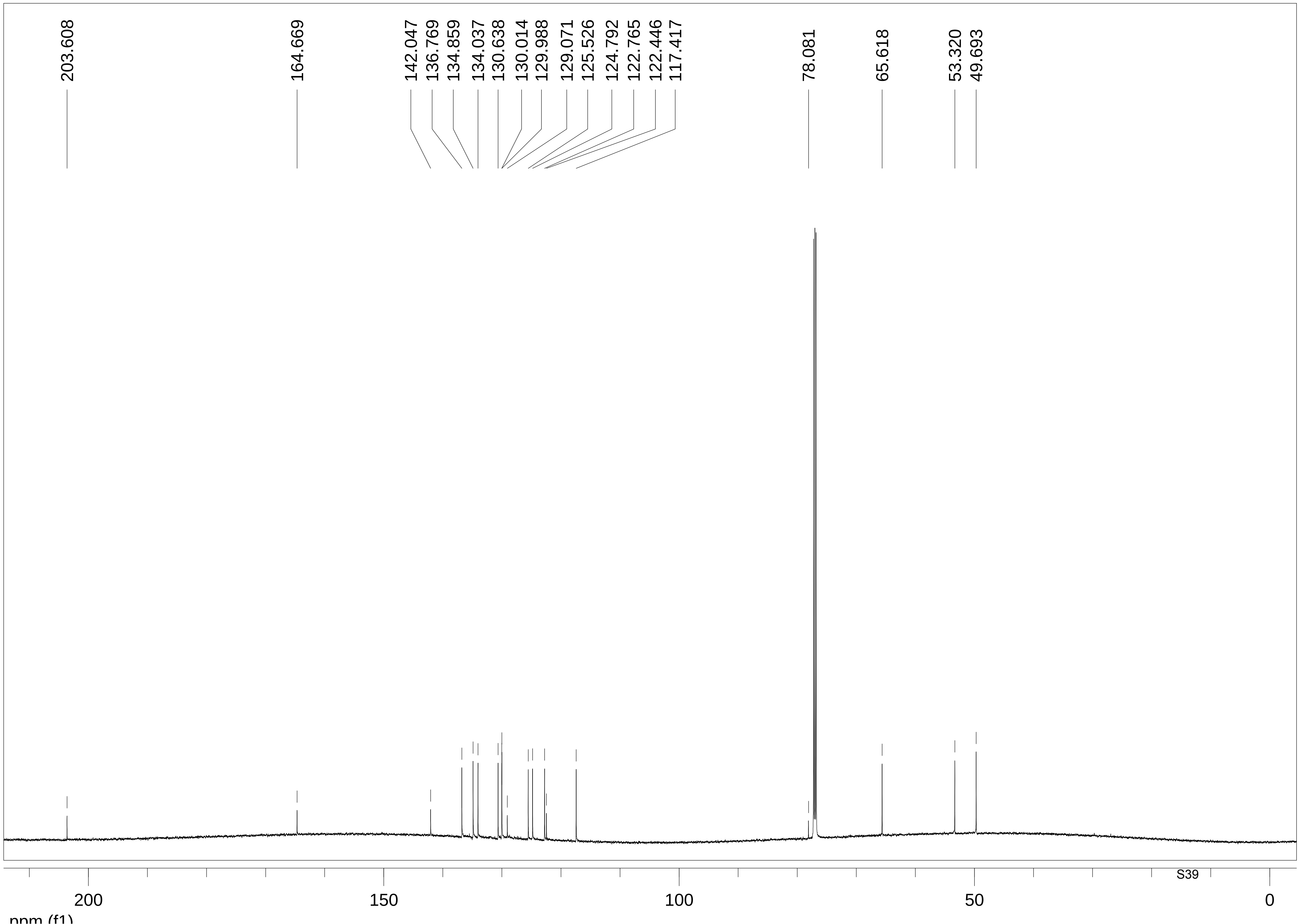
203.608
164.669
142.047
136.769
134.859
134.037
130.638
130.014
129.988
129.071
125.526
124.792
122.765
122.446
117.417
78.081
65.618
53.320
49.693
200
150
100
50
0
ppm (f1)

Chromatogram : SR_592rac_ADH_9010_flow06_acq608

Data file: SR_592rac_ADH_9010_flow06_acq608.DATA
Method: HPLC2_ADH_9010_flow06_acq60
Date: 11.11.2011 18:18:14

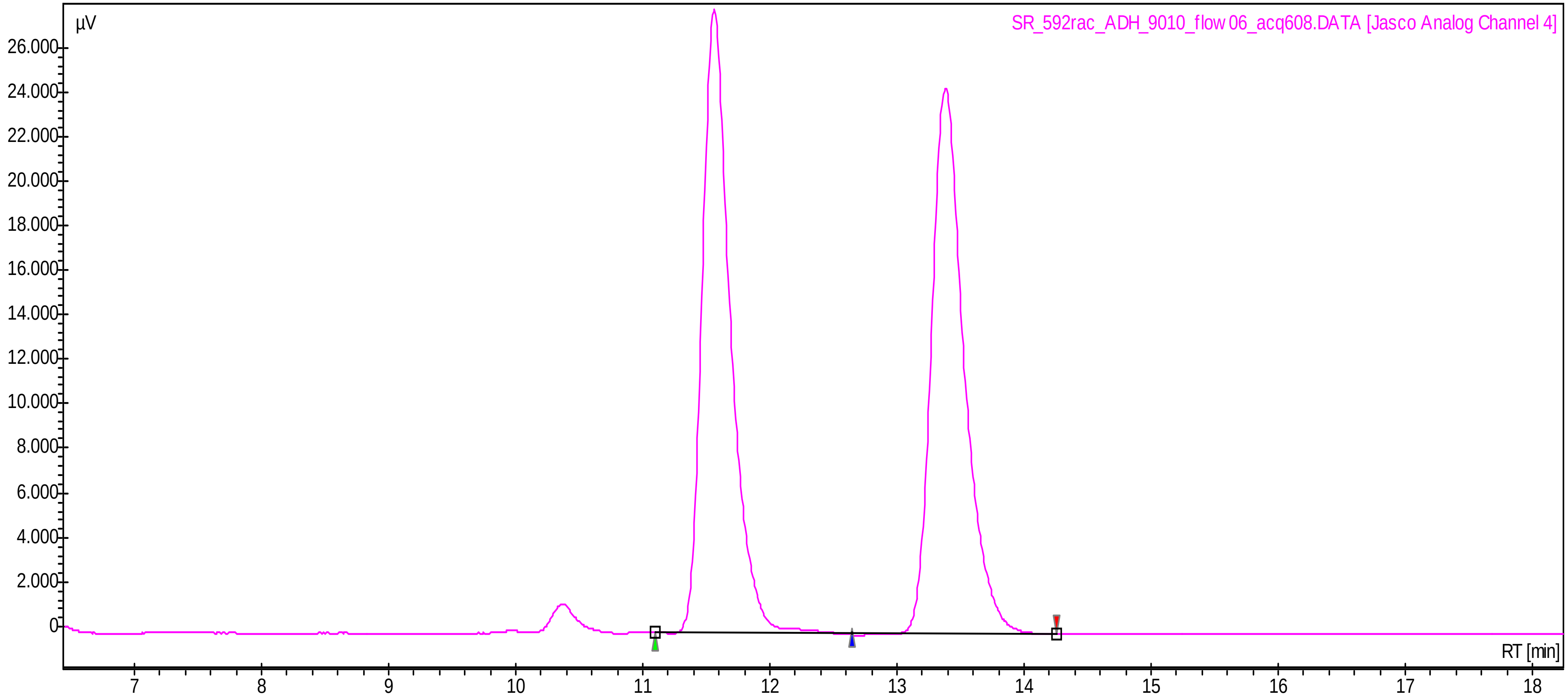

SR_592rac_ADH_9010_flow06_acq608.DATA [Jasco Analog Channel 4]

Index	Start	Time	End	Area %
	[Min]	[Min]	[Min]	[%]
1	11,095	11,567	12,645	49,838
2	12,645	13,383	14,256	50,162
Total				100,000

Chromatogram : SR_592.2 c_ADH_9010_flow06_acq302

Data file: SR_592.2 c_ADH_9010_flow06_acq302.DATA
Method: HPLC2_ADH_9010_flow06_acq30
Date: 30.11.2011 10:29:52

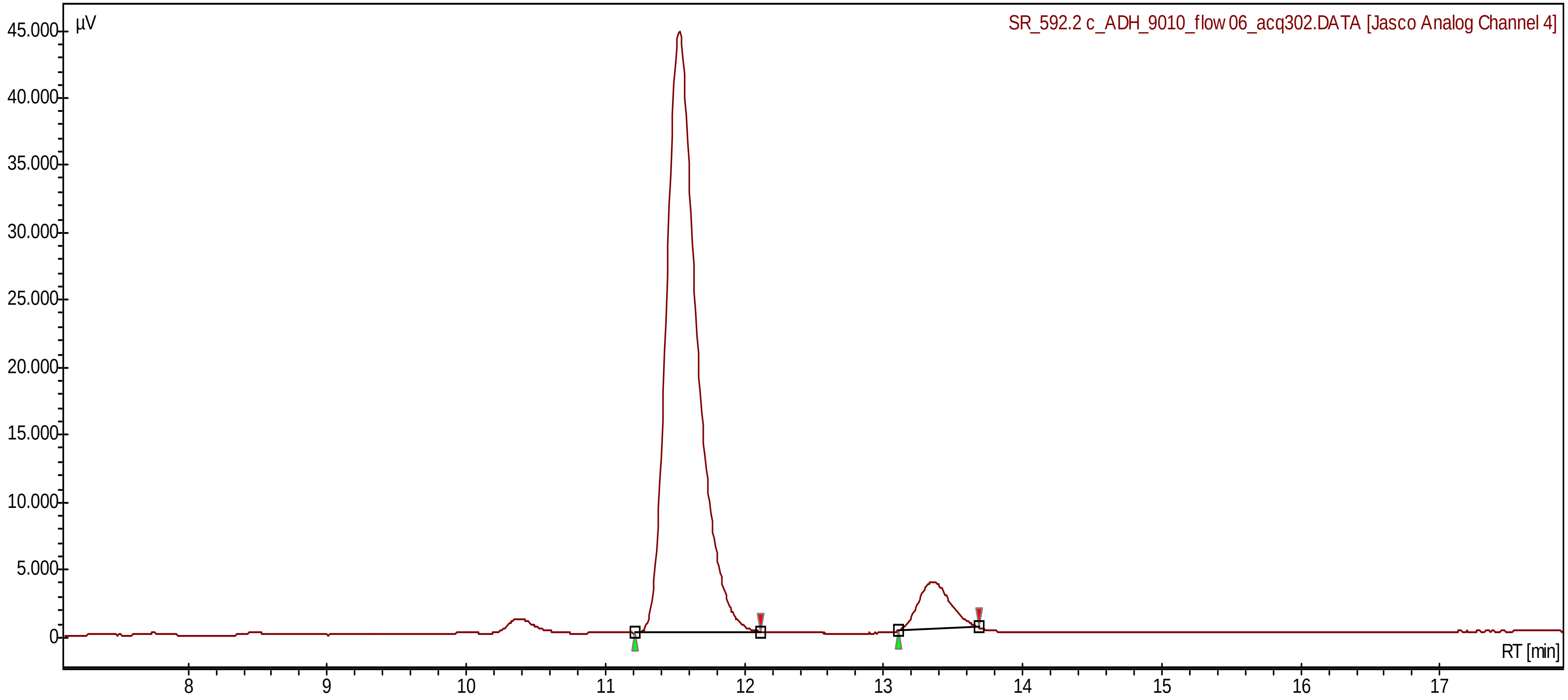

SR_592.2 c_ADH_9010_flow06_acq302.DATA [Jasco Analog Channel 4]

Index	Start	Time	End	Area %
	[Min]	[Min]	[Min]	[%]
1	11,214	11,533	12,111	92,399
2	13,107	13,350	13,686	7,601
Total				100,000

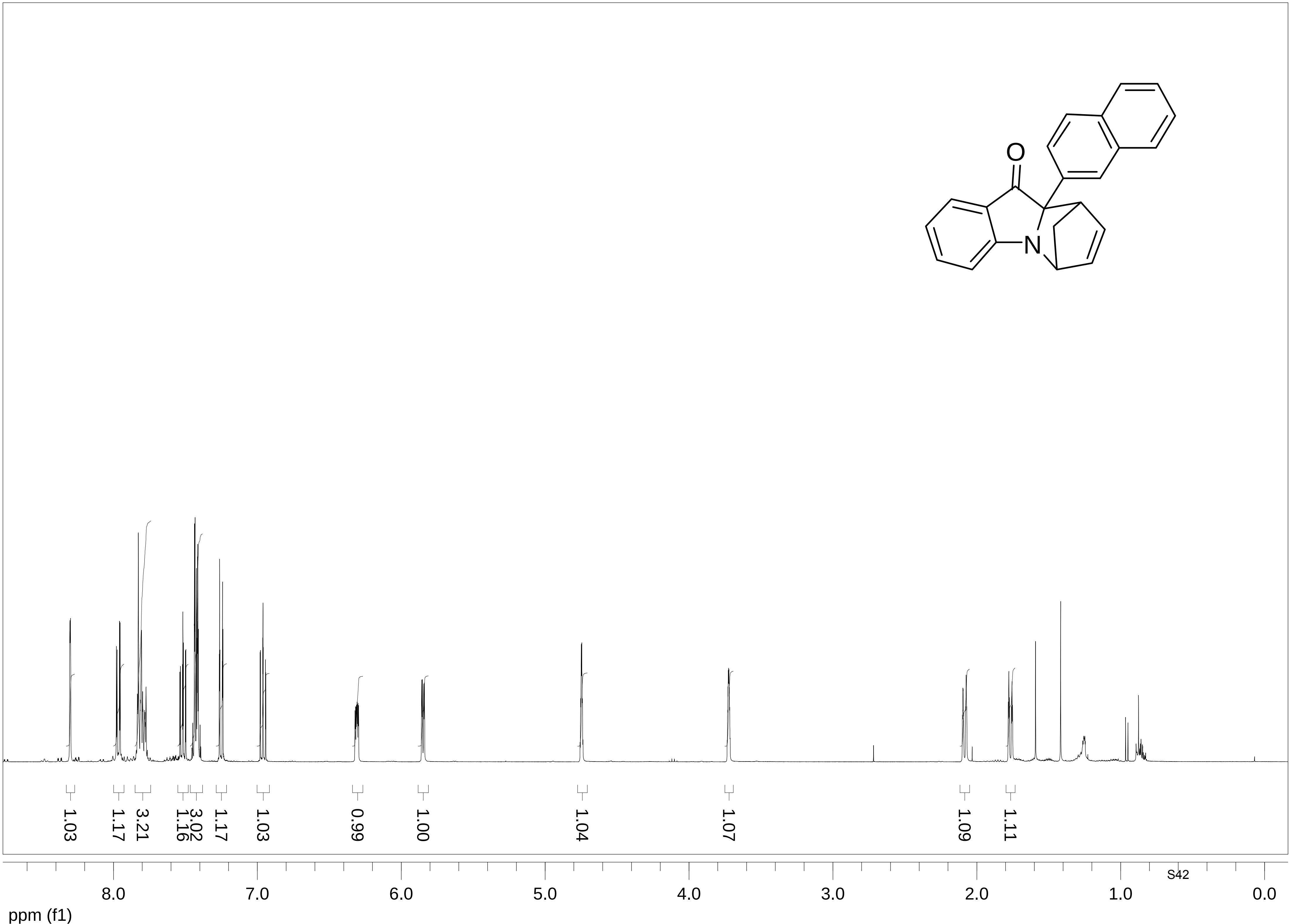
O
N
1.03
1.17
3.21
1.16
3.02
1.17
1.03
0.99
1.00
1.04
1.07
1.09
1.11
8.0
7.0
6.0
5.0
4.0
3.0
2.0
1.0
0.0
ppm (f1)

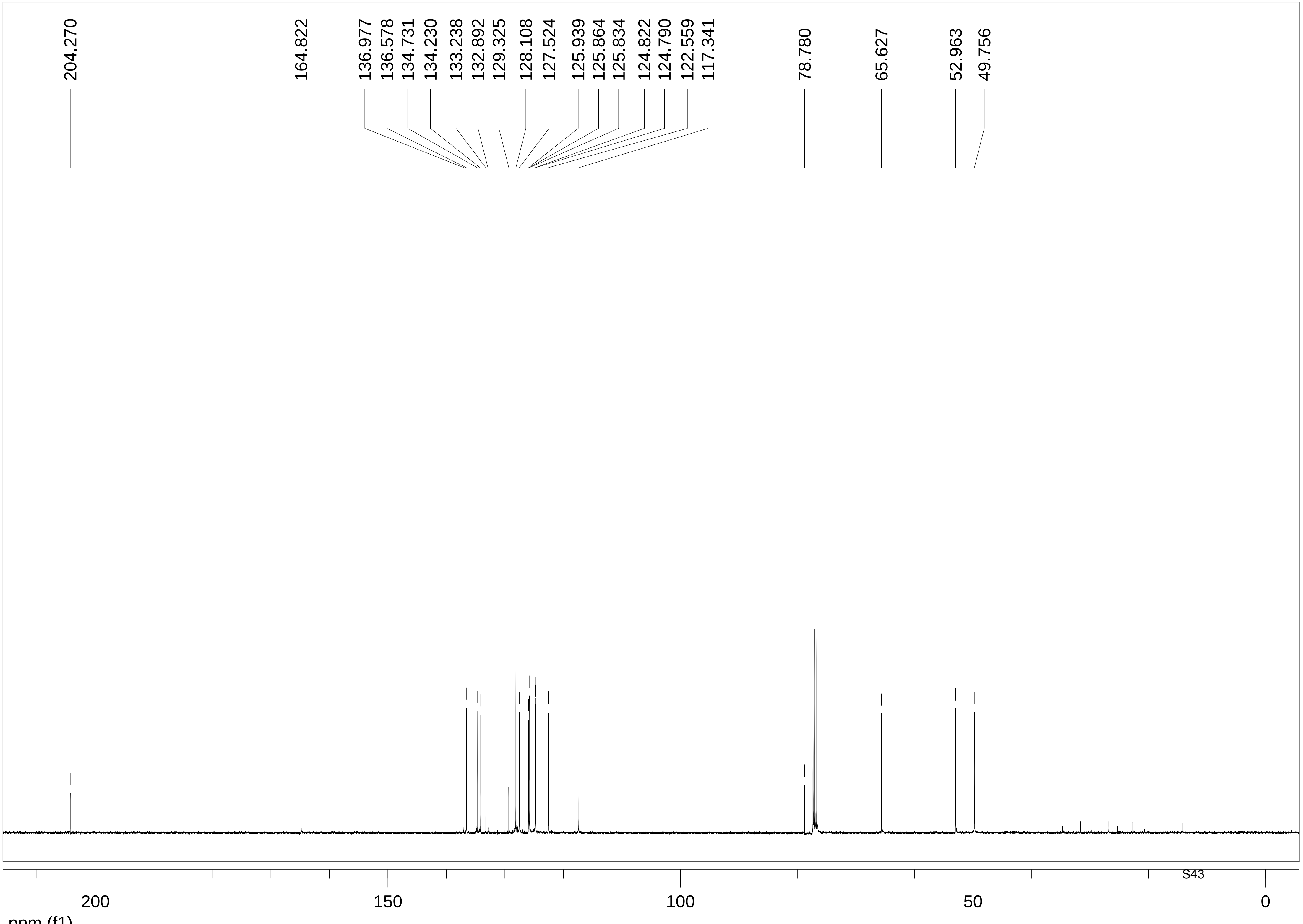
204.270
164.822
136.977
136.578
134.731
134.230
133.238
132.892
129.325
128.108
127.524
125.939
125.864
125.834
124.822
124.790
122.559
117.341
78.780
65.627
52.963
49.756
200
150
100
50
0
ppm (f1)

Chromatogram : SR_593_rac_ADH_9010_flow06_acq609

Data file: SR_593_rac_ADH_9010_flow06_acq609.DATA
Method: HPLC2_ADH_9010_flow06_acq60
Date: 17.11.2011 16:41:28

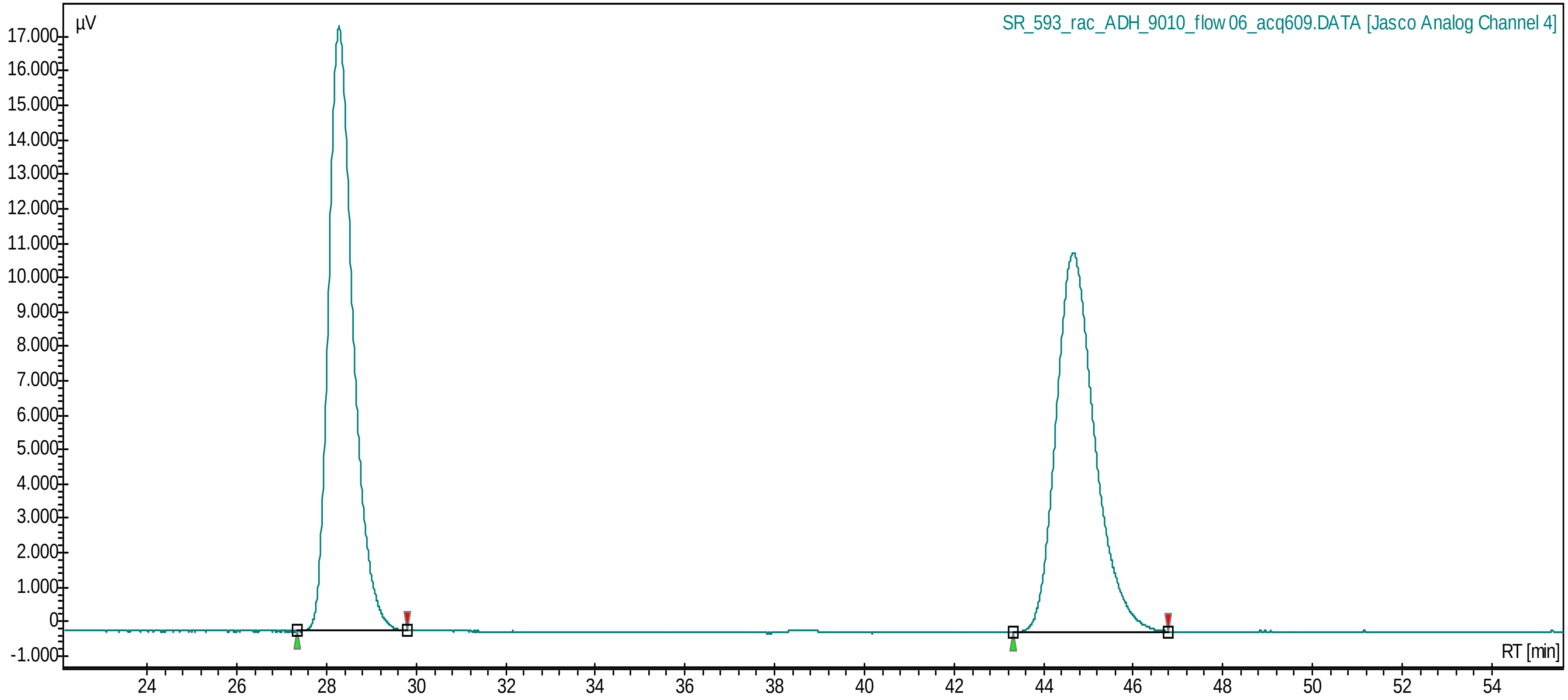

SR_593_rac_ADH_9010_flow06_acq609.DATA [Jasco Analog Channel 4]

Index	Start	Time	End	Area %
	[Min]	[Min]	[Min]	[%]
1	27,335	28,283	29,814	50,195
2	43,326	44,667	46,798	49,805
Total				100,000

Chromatogram : SR_593.3_c_ADH_9010_flow06_acq6013

Data file: SR_593.3_c_ADH_9010_flow06_acq6013.DATA
Method: HPLC2_ADH_9010_flow06_acq60
Date: 30.11.2011 20:28:05

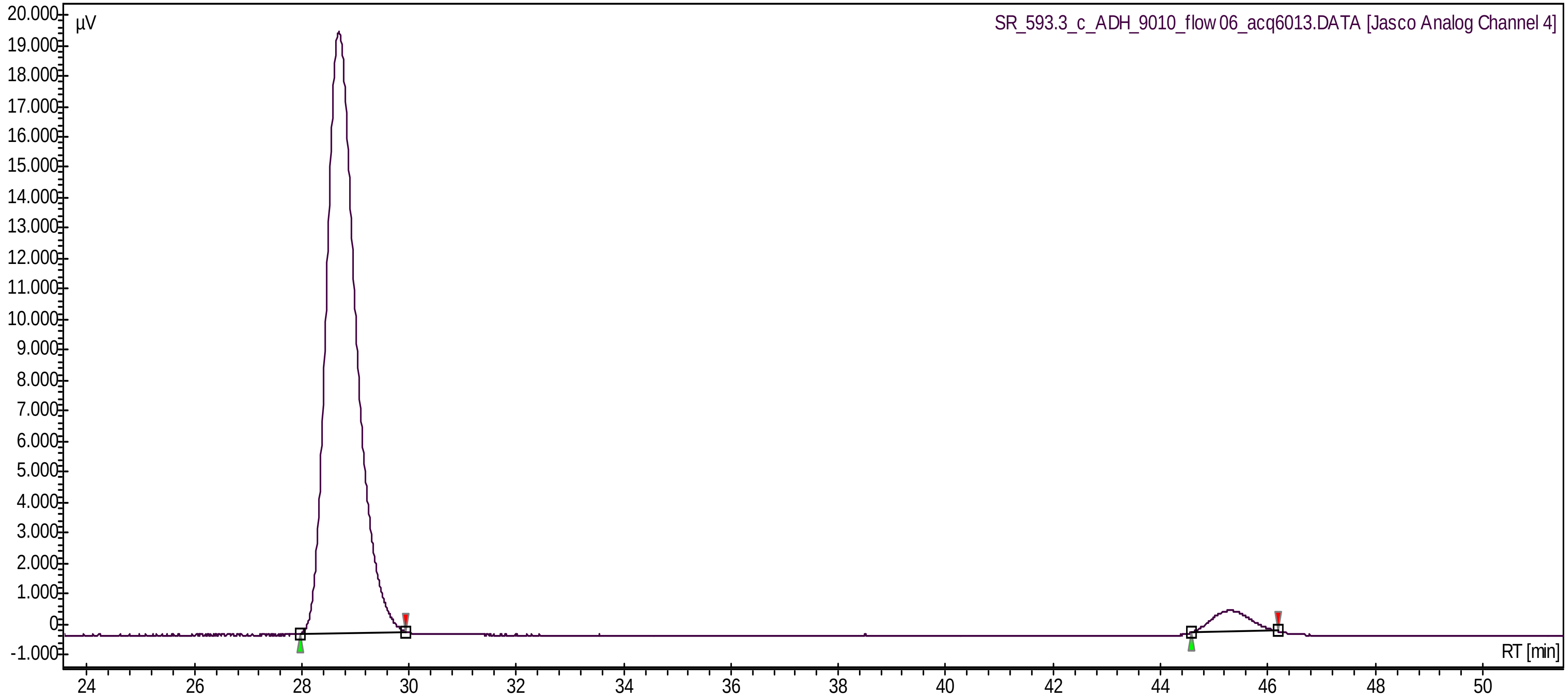

SR_593.3_c_ADH_9010_flow06_acq6013.DATA [Jasco Analog Channel 4]

Index	Start	Time	End	Area %
	[Min]	[Min]	[Min]	[%]
1	27,960	28,692	29,948	95,611
2	44,586	45,300	46,199	4,389
Total				100,000

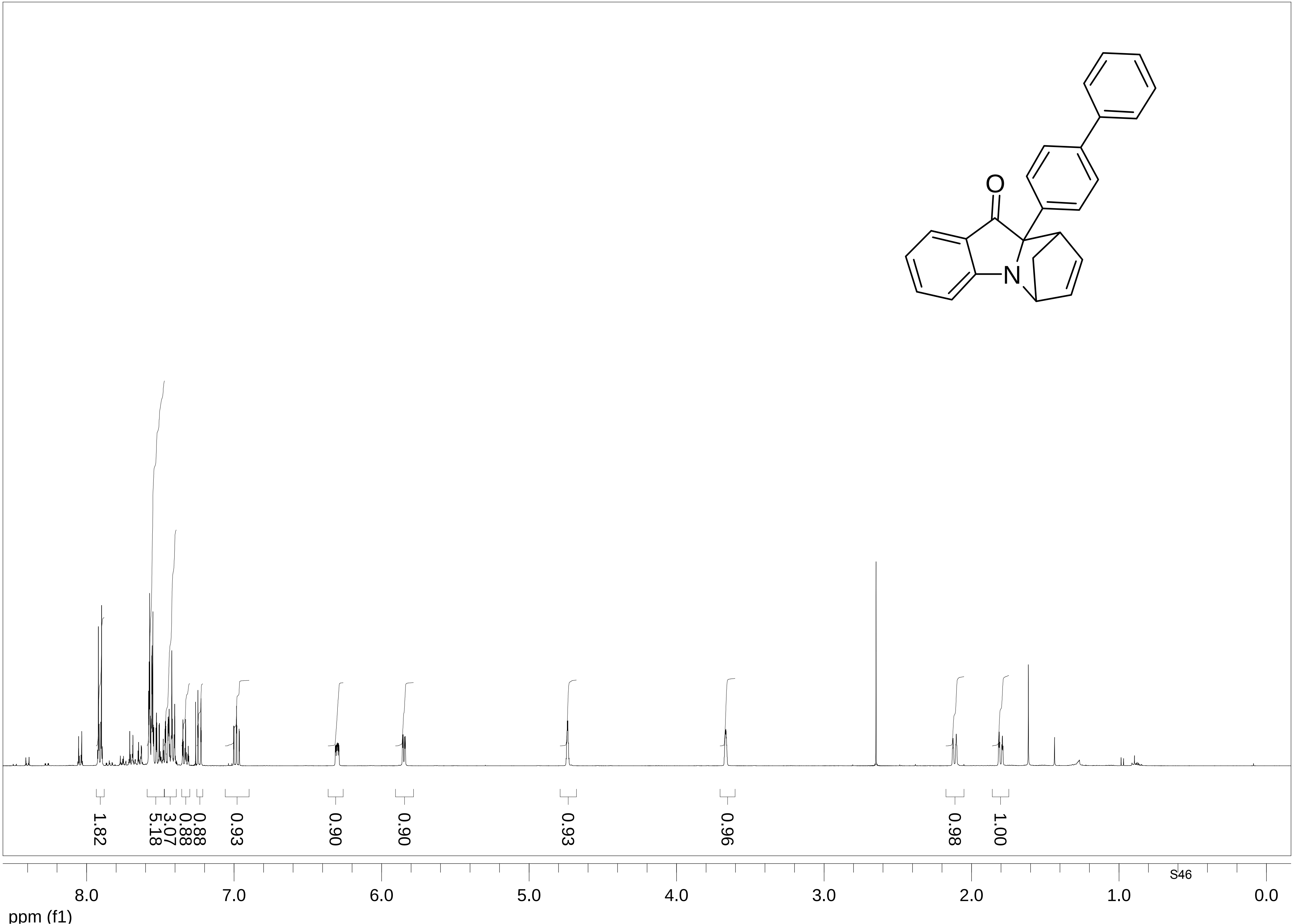
O
N
1.82
5.18
3.07
0.88
0.88
0.93
0.90
0.90
0.93
0.96
0.98
1.00
8.0
7.0
6.0
5.0
4.0
3.0
2.0
1.0
0.0
ppm (f1)

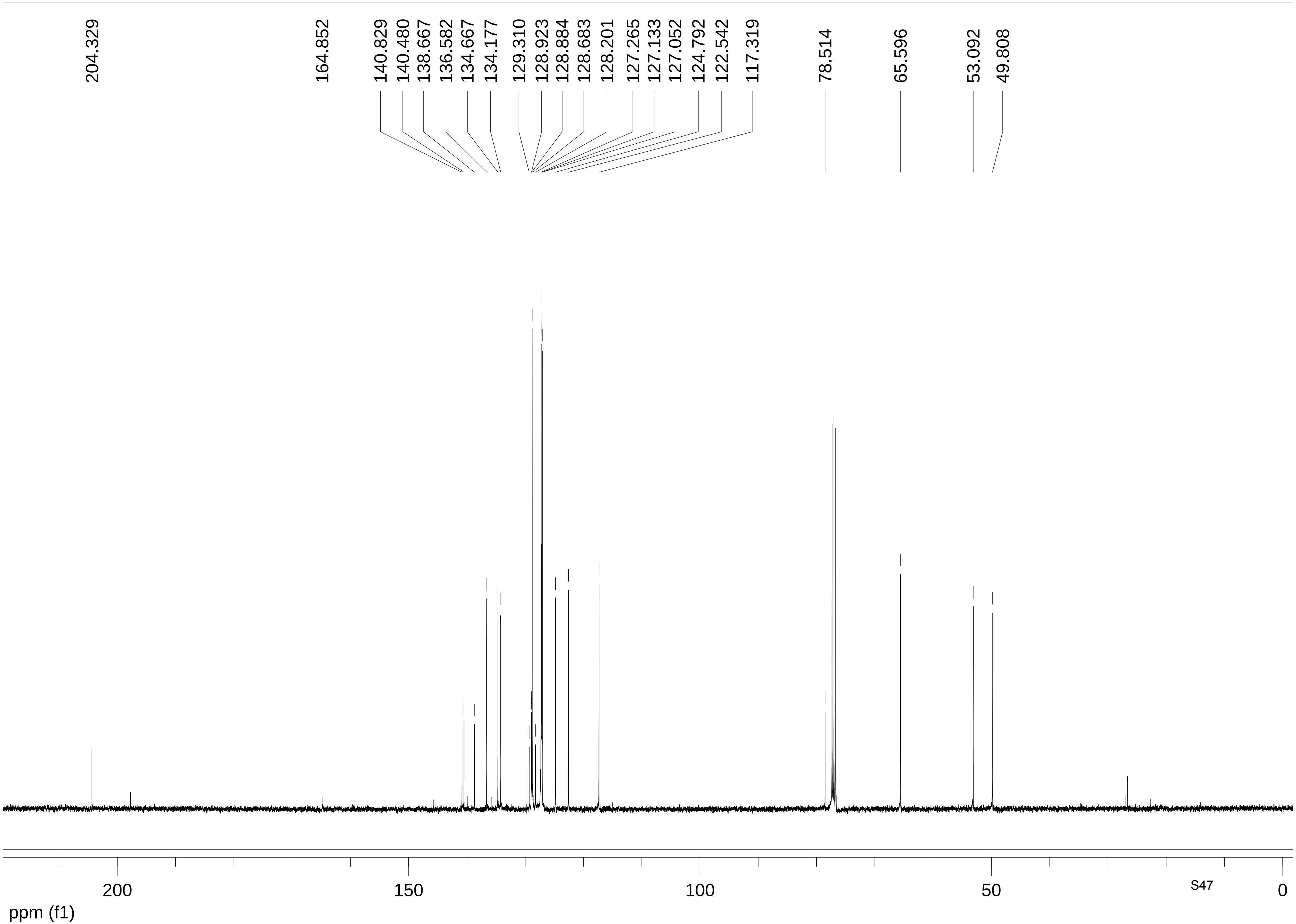
204.329
164.852
140.829
140.480
138.667
136.582
134.667
134.177
129.310
128.923
128.884
128.683
128.201
127.265
127.133
127.052
124.792
122.542
117.319
78.514
65.596
53.092
49.808
200
150
100
50
0
ppm (f1)

Chromatogram : SR_594_rac_ADH_9010_flow06_acq603

Data file: SR_594_rac_ADH_9010_flow06_acq603.DATA
Method: HPLC2_ADH_9010_flow06_acq50
Date: 30.11.2011 10:50:22

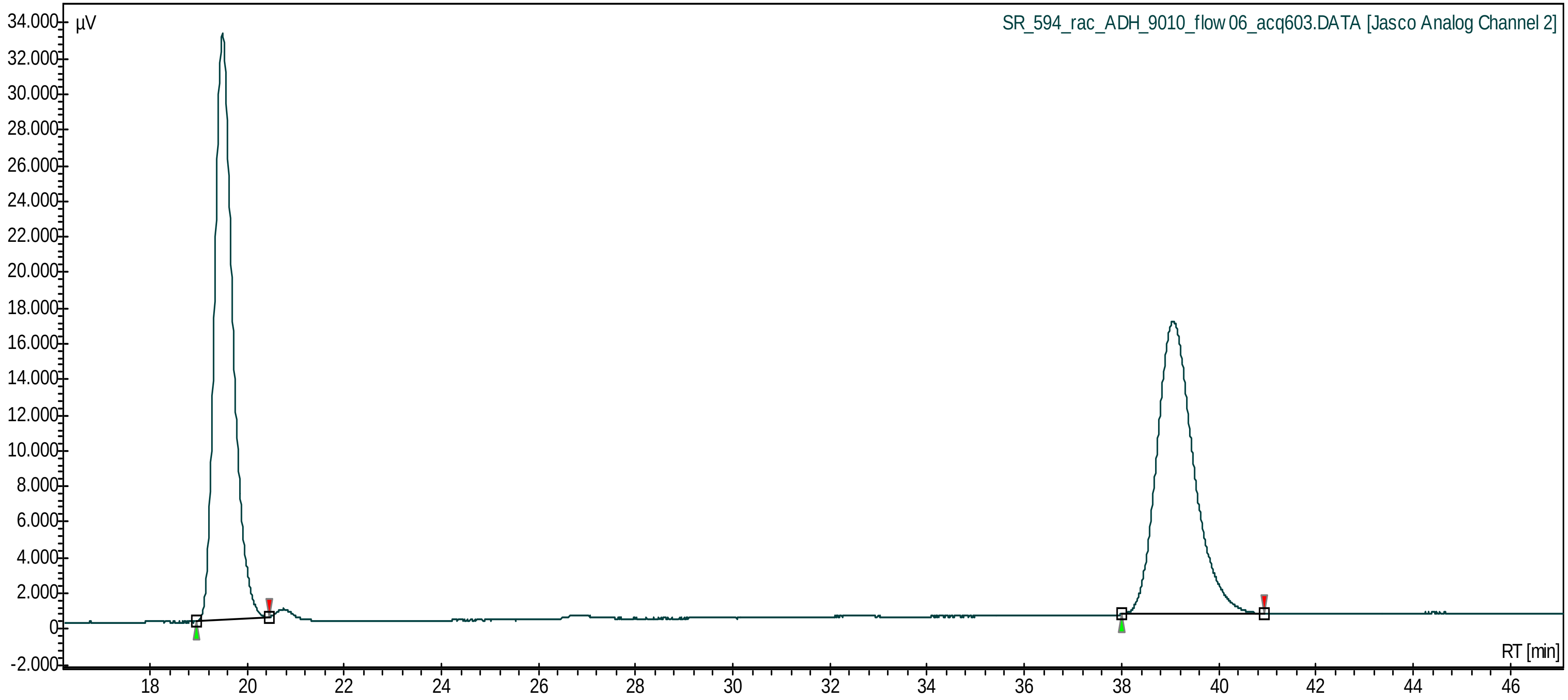

SR_594_rac_ADH_9010_flow06_acq603.DATA [Jasco Analog Channel 2]

Index	Start	Time	End	Area %
	[Min]	[Min]	[Min]	[%]
1	18,956	19,500	20,470	49,798
2	38,009	39,067	40,941	50,202
Total				100,000

Chromatogram : SR_594_c_ADH_9010_flow06_acq604

Data file: SR_594_c_ADH_9010_flow06_acq604.DATA
Method: HPLC2_ADH_9010_flow06_acq50
Date: 30.11.2011 11:40:07

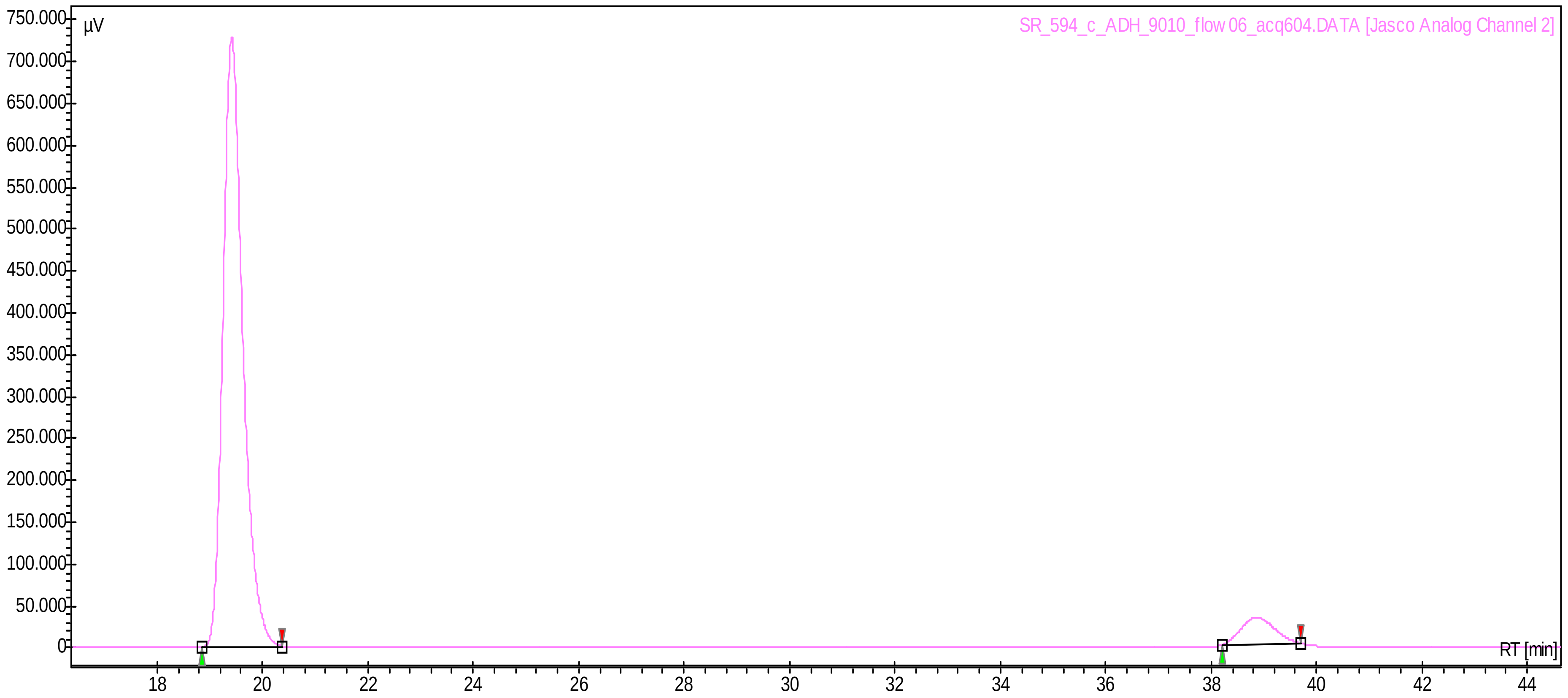

SR_594_c_ADH_9010_flow06_acq604.DATA [Jasco Analog Channel 2]

Index	Start	Time	End	Area %
	[Min]	[Min]	[Min]	[%]
1	18,855	19,417	20,373	93,112
2	38,207	38,858	39,695	6,888
Total				100,000
